# Transposable Elements Contribute to the Regulation of Long Noncoding RNAs in *Drosophila melanogaster*

**DOI:** 10.3390/insects15120950

**Published:** 2024-11-30

**Authors:** Yuli Gan, Lingyan Wang, Guoxian Liu, Xiruo Guo, Yiming Zhou, Kexin Chang, Zhonghui Zhang, Fang Yan, Qi Liu, Bing Chen

**Affiliations:** 1 College of Life Science, Hebei University, Baoding 071002, China; ganyuli123@163.com (Y.G.); wly1372@163.com (L.W.); guoxiruo@foxmail.com (X.G.); 2 Rice Research Institute, Guangdong Academy of Agricultural Sciences, Guangzhou 510640, China; guoxianliu0205@163.com; 3 Guangdong Provincial Key Laboratory of Biotechnology for Plant Development, School of Life Science, South China Normal University, Guangzhou 510631, China; 15137361521@163.com (Y.Z.); zhzhang@m.scnu.edu.cn (Z.Z.); 4 Key Laboratory of Herbage and Endemic Crop Biotechnology, Ministry of Education, School of Life Science, Inner Mongolia University, Hohhot 010021, China; changkexincas@163.com (K.C.); yanfang@imu.edu.cn (F.Y.); 5 Hebei Basic Science Center for Biotic Interaction, Hebei University, Baoding 071002, China

**Keywords:** long noncoding RNA, transposable element, *Drosophila*, TE-lncRNA, heavy metal

## Abstract

**Background**: Transposable elements (TEs) and noncoding sequences are major components of the genome, yet their functional contributions to long noncoding RNAs (lncRNAs) are not well understood. Although many lncRNAs originating from TEs (TE-lncRNAs) have been identified across various organisms, their characteristics and regulatory roles, particularly in insects, remain largely unexplored. This study integrated multi-omics data to investigate TE-lncRNAs in *D. melanogaster*, focusing on the influence of transposons across different omics levels. **Results**: We identified 16,118 transposons overlapping with lncRNA sequences that constitute 2119 TE-lncRNAs (40.4% of all lncRNAs) using 256 public RNA-seq samples and 15 lncRNA-seq samples of *Drosophila* S2 cells treated with heavy metals. Of these, 67.2% of TE-lncRNAs contain more than one TE. The LTR/Gypsy family was the most common transposon insertion. Transposons preferred to insert into promoters, transcription starting sites, and intronic regions, especially in chromosome ends. Compared with lncRNAs, TE-lncRNAs showed longer lengths, a lower conservation, and lower levels but a higher specificity of expression. Multi-omics data analysis revealed positive correlations between transposon insertions and chromatin openness at the pre-transcriptional level. Notably, a total of 516 TE-lncRNAs provided transcriptional factor binding sites through transposon insertions. The regulatory network of a key transcription factor was rewired by transposons, potentially recruiting other transcription factors to exert regulatory functions under heavy metal stress. Additionally, 99 TE-lncRNAs were associated with m^6^A methylation modification sites, and 115 TE-lncRNAs potentially provided candidate small open reading frames through transposon insertions. **Conclusions**: Our data analysis demonstrated that TEs contribute to the regulation of lncRNAs. TEs not only promote the transcriptional regulation of lncRNAs, but also facilitate their post-transcriptional and epigenetic regulation.

## Simple Summary

This study focused on lncRNAs originating from TEs (TE-lncRNAs) in *Drosophila melanogaster* by integrating multi-omics data. We identified 2119 TE-lncRNAs (40.4% of all lncRNAs) using 271 RNA-seq, of which the LTR/Gypsy family was the most common transposon. In addition, transposons preferred certain genic regions. TE-lncRNAs had longer lengths, a lower conservation, and a specific expression. Multi-omics analysis showed positive correlations between transposon insertions and chromatin openness; some TE-lncRNAs provided transcription factor binding sites, rewired regulatory networks, and provided candidate small open reading frames through TE insertions. Thus, TEs contribute to lncRNAs, promoting transcriptional, post-transcriptional, and epigenetic regulation.

## 1. Introduction

Approximately 98% of the human genome is transcribed into noncoding RNAs [1], among which long noncoding RNAs (lncRNAs) constitute a significant portion of noncoding RNA molecules. LncRNAs are characterized by a low-coding potential and a length exceeding 200 nucleotides; they are broadly categorized into intronic lncRNA, intergenic lncRNA, and antisense lncRNA [2]. LncRNAs play diverse roles at different molecular levels, including transcriptional interference, chromatin remodeling and nucleosome modification, alternative splicing regulation, endogenous siRNA biogenesis, control of protein activity, etc. [3]. At the post-transcriptional level, lncRNAs contain m^6^A modification sites related to disease [4,5] and small open reading frames (sORFs) that are often overlooked by traditional gene annotation methods. These sORFs can encode short peptides and some lncRNAs are even considered “dual-functional” RNAs, exerting regulatory roles in their RNA form and functional roles through peptide coding [6,7]. Several lncRNA-encoded short peptides have been discovered, such as SPAR [8], Minion/myomixer [9], HOXB-AS3 [10], NOBODY [11], and APPLE [12] in humans; MLN [13], and DWORF [14] in mice; Toddler [15] in zebrafish; and Tarsal-less/tal [16], Scl [17], and Pgc [18] in fruit flies, demonstrating significant regulatory functions. Recent analysis have revealed that lncRNAs play a significant role through the regulation of transposon activities [19]. Despite their diverse functions, the role of lncRNAs remains underexplored.

Transposable elements (TEs) are DNA sequences that are capable of being excised from their original location, replicated or not, and inserted elsewhere, thereby affecting adjacent genes. TEs are broadly classified into two categories: class I transposons, also known as retrotransposons, which employ a “copy and paste” mechanism, such as LTR, LINE, and SINE; and class II transposons, or DNA transposons, which utilize a “cut and paste” mechanism, of which there are two types of subclasses, subclass 1, e.g., TIR, and subclass 2, e.g., Helitrons, which is a recently discovered class of DNA transposons that transpose via a rolling-circle mechanism [20,21,22,23,24]. TEs constitute a 22% portion of genomes in *Drosophila melanogaster* [25] and 66% in humans [26]. Although TEs are often considered as “junk DNA”, many of them also play various crucial roles in gene expression regulation and phenotypic evolution [26,27,28]. For example, Gypsy insertion in ducks caused bodyweight enlargement or white plumage phenotype formation, while the insertion of Alu elements led to the loss of their tail in humans [29,30]. Studies have highlighted the role of transposons in promoting the nuclear enrichment of lncRNAs, indicating their essential contribution to lncRNA functionality [31]. However, the intricate relationship between transposons and lncRNAs remains elusive.

Many lncRNAs overlap with TE sequences or originate from TEs, and are regarded as TE-lncRNAs. In recent years, numerous TE-lncRNAs have been identified and characterized across various species [32,33,34]. These TE-lncRNAs are widespread in both animals and plants [35,36,37]. The emergence and evolution of lncRNAs are closely intertwined with TEs [33,38]. However, the nature and regulatory functionality of TE-lncRNAs, especially in insects, largely remain elusive. Our understanding of the characteristics and features of TE-lncRNAs, as well as the impact of transposons on lncRNAs at different regulatory layers, is limited. A comprehensive investigation spanning transcriptional and post-transcriptional layers is imperative to elucidate the intricate mechanisms by which transposons shape the biogenesis and functional roles of lncRNAs.

Here, we hypothesized that TEs in lncRNAs contribute to biogenesis and regulatory functionality of lncRNAs. *D. melanogaster* serves as a model organism within the insect kingdom. This study comprehensively identified and analyzed the characteristics of TE-lncRNAs in *D. melanogaster* and explored TE-lncRNAs at the epigenetic, transcriptional, and post-transcriptional levels, investigating the contribution of transposons in the biogenesis, characteristics, and functional roles of lncRNAs across these dimensions. Furthermore, the functional roles of TE-lncRNAs under stress conditions, such as heavy metal exposure, were explored. Thus, this research discovered the significance of transposons and noncoding sequences, which have previously dismissed as “junk sequences” or “dark matter,” and established the intricate connection between transposons and noncoding sequences at multiple regulatory levels.

## 2. Materials and Methods

### 2.1. Data

A total of 256 RNA-seq samples were collected from the Berkeley *Drosophila* Genome Project (BDGP), with the NCBI accession ID SRA009364 [39,40]. A further 15 lncRNA-seq datasets from heavy metal-treated *Drosophila* S2 cells were sequenced by our lab [41]. Low-quality samples were filtered out, and all datasets consisted of paired-end reads. Detailed sample information is provided in Supplementary Table S2. Assay for Transposase-Accessible Chromatin with high-throughput sequencing (ATAC-seq) data from 28 *D. melanogaster* samples were downloaded from NCBI, with the corresponding sample information being listed in Supplementary Table S3. Methylated RNA Immunoprecipitation Sequencing (MeRIP-seq) data from 8 *D. melanogaster* samples were also obtained from NCBI, with details being provided in Supplementary Table S4. Furthermore, ribosome profiling sequencing (Ribo-seq) data from 72 *D. melanogaster* samples were downloaded from NCBI, and the associated sample information can be found in Supplementary Table S5. Lastly, *D. melanogaster* proteomics data were acquired from ProteomeXchange under the project ID PXD021022.

### 2.2. LncRNA Identification Pipeline

The datasets were downloaded and converted to FASTQ format using sratoolkit. The reads were then cleaned with fastp [42] and mapped to the reference genome (*D. melanogaster*, Release 6 plus ISO1 MT, NCBI) using HISAT2 [43]. The mapped reads were assembled and merged with StringTie [44] to generate a merged GTF file, which was then compared to the reference annotation using GffCompare [45]. StringTie compares the assembled transcripts against the reference gene annotations (usually in a GTF file). It assesses the transcript’s exon boundaries, splice sites, genomic coordinates, and strand information to identify different types of transcripts. If there is no continuous splice junction information between exons, StringTie is more likely to treat these exons as belonging to different transcripts, avoiding misassembly. The transcripts were filtered through the following three steps: (i) retaining only those annotated with class codes u, x, i, j, or o, where “i”: fully contained within a reference intron, “j”: multi-exon with at least one junction match, “o”: other same-strand overlap with reference exons, “x”: exonic overlap on the opposite strand, “u”: unknown and intergenic; (ii) ensuring transcript length was at least 200 bp; and (iii) including only transcripts with at least one exon. For coding potential filtering, we employed a three-step process as follows: first, the coding potential was predicted using CNCI and CPC2 [46], and the results were intersected to identify noncoding sequences; second, these sequences were mapped to *D. melanogaster* protein sequences using BLAST [47], filtering out protein-coding sequences to identify candidate lncRNAs; third, the expression levels of candidate lncRNAs were calculated using FeatureCounts [48] and R scripts, retaining only those expressed in at least one sample. For each lncRNA, we analyzed its expression across all 271 samples. If the total sum of FPKM values for a given lncRNA across these samples was greater than zero, we retained that lncRNA, considering it to be expressed in at least one of the samples.

### 2.3. TE Annotation

TEs were annotated for genome (*D. melanogaster* reference genome, Release 6 plus ISO1 MT, NCBI), CDS, and lncRNA sequences using RepeatMasker [49,50], and RepBase 20181026 [51,52] as the library. The following parameters were used: -libdir Libraries -species “*Drosophila melanogaster*” -e ncbi -nolow -pa 20. The low-complexity and simple repeat sequences were removed; only the TE sequences were retained. Then, makeTEGTF.pl was used to convert the output file of RepeatMasker into gtf format; the following parameters were used: -c 5 -s 6 -e 7 -o 9 -t 10 -f 11 -S. When the lncRNA sequence overlapped with the TE sequence, it was regarded as a TE-lncRNA.

### 2.4. General Bioinformatical Analysis

The ChIPseeker [53] R package was used to statistically analyze and visualize the positional distribution of TE insertions, coding genes, TE-lncRNAs, and Non-TE-lncRNAs. Conservation was assessed using PhyloP and PhastCons scores, which were obtained from the “dm6.phyloP124way” and “dm6.phastCons124way” datasets. The score values refer to evolutionary conservation scores based on a multiple sequence alignment of the fruit fly genome (*D. melanogaster*, version dm6) with a set of 124 species. These species are primarily chosen for their evolutionary proximity to *D. melanogaster*, providing insight into both functional conservation and evolutionary pressures. The 124 species typically include *Drosophila* species, as well as close relatives such as *Drosophila simulans*, *Drosophila yakuba*, *Drosophila pseudoobscura*, etc. All the 124 species are shown in the txt file from UCSC (http://hgdownload.soe.ucsc.edu/goldenPath/dm6/phyloP124way/assemblyInformation.txt, accessed on 29 November 2018). The levels and specificity of gene expression were analyzed using TBtools [54]. The TAU (tissue-specific gene expression) index indicates how specific or broadly expressed a gene or transcript is within studied tissues [55]. TBtools uses the expression matrix of genes and lncRNAs as input data to compute the TAU index for each gene and lncRNA. The TAU index has a range of 0–1, whereby a value closer to 1 indicate more expression specificity. The expression specificity calculation formula is $\tau =\frac{\sum_{i=1}^{N}\left(1-x_{i}\right)}{N-1}$. We also constructed a co-expression network using the WGCNA [56] R package. Finally, coding genes were selected as input data for Gene Ontology (GO) functional enrichment analysis, which was performed using the clusterProfiler [57] R package.

### 2.5. ATAC-Seq Analysis

ATAC-seq data were downloaded and extracted using sratoolkit to obtain data in FASTQ format. Quality control was performed using fastp; then, the quality-controlled data were aligned to the reference genome (*D. melanogaster*, Release 6 plus ISO1 MT, NCBI) using STAR. The alignment results (BAM files) were converted to bigWig (BW) files using deepTools [58], and the data were visualized using IGV. Chromatin-accessible regions were identified by calling peaks with MACS2 [59] based on the alignment results, and BEDTools [60] was used to intersect lncRNA locations with ATAC-seq peak regions to determine the chromatin accessibility of lncRNAs.

### 2.6. MeRIP-Seq Analysis

MeRIP-seq data were downloaded and extracted using sratoolkit to obtain data in FASTQ format. Quality control was performed using fastp; then; the quality-controlled data were aligned to the fruit fly reference genome (*D. melanogaster*, Release 6 plus ISO1 MT, NCBI) using STAR. Based on the alignment results (BAM files), the R packages exomePeak2 [61] and RNAmod [62] were used to identify m^6^A modification sites. Then, BEDTools was used to intersect the locations of lncRNAs with m^6^A peak regions to determine the m^6^A methylation modification sites on lncRNAs.

### 2.7. Ribo-Seq Analysis

First, candidate ORFs were annotated within lncRNAs; then, these annotations with Ribo-seq data were integrated to identify translated ORFs within lncRNAs. The Ribo-seq data were downloaded from NCBI using sratoolkit, which was then extracted into FASTQ format, and preprocessed by removing adapters with cutadapt, filtering with the fastq_quality_filter, and converting to FASTA format. Next, Bowtie [63] and SAMtools [64] were used to remove tRNA and rRNA sequences; then, the cleaned data were aligned with STAR. Finally, translated lncRNAs were identified and verified using tools such as Ribo-TISH [65], riboWaltz [66], ribORF [67], and RiboToolkit [68] and were quantified using FeatureCounts. When we ran the pipeline of the Ribo-seq, we set the length at 26–34 RPF.

### 2.8. Proteomic Analysis

Using the annotated candidate ORFs on lncRNAs as a database, a database search analysis was performed on the downloaded raw proteomic mass spectrometry files using MaxQuant [69]. Based on the search results, the peptide information encoded by ORFs and secondary mass spectra of the peptides was visualized using PDV [70].

## 3. Results

### 3.1. Identification of lncRNAs and TEs in D. melanogaster

We compiled a total of 271 transcriptome datasets, distributed across various tempo-spatial scales and experimental conditions in *D. melanogaster* (Table S1). These datasets comprised 256 paired-end RNA-seq datasets from the BDGP (Berkeley *Drosophila* Genome Project) database, and 15 paired-end lncRNA-seq datasets generated in our laboratory from *Drosophila* S2 cells exposed to four distinct heavy metal treatments (Cd, Cu, Pb, and Zn) [41].

**Figure 1:**
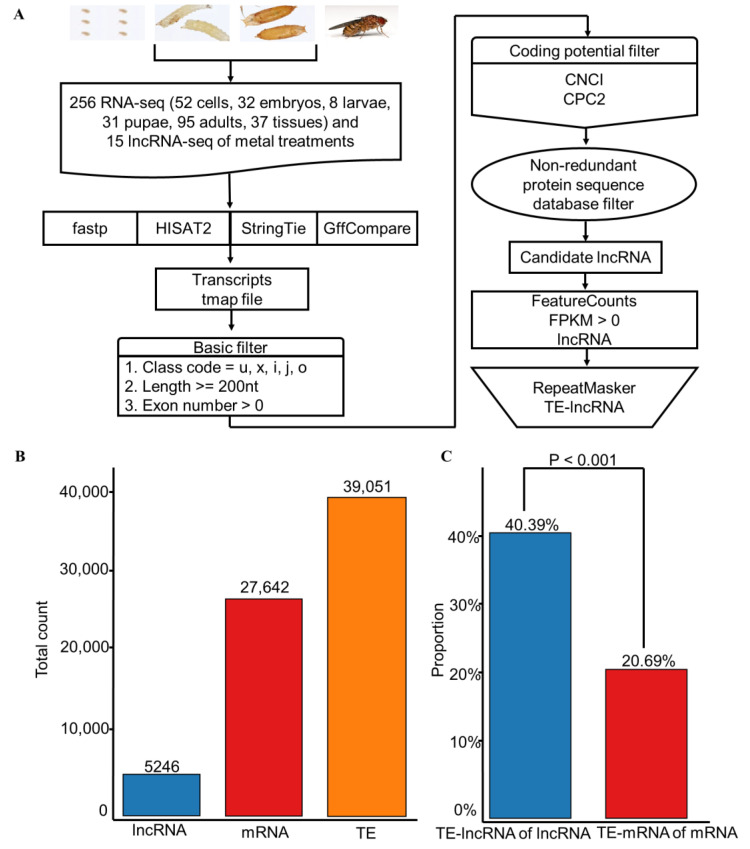
The pipeline of identifying TE-lncRNAs from RNA-seq data. (**A**) The pipeline of identifying TE-lncRNAs. (**B**) A total of 27,642 mRNAs, 5246 lncRNAs, and 39,051 TEs were identified in *D. melanogaster*. (**C**) The proportion of TE-lncRNAs in lncRNAs and TE-mRNAs in mRNAs.

We established a robust pipeline tailored to the identification of TE-lncRNAs in *D. melanogaster*, delineated into three key steps (Figure 1A). First, we rigorously cleaned the 271 RNA-seq datasets using fastp [42], and aligned them to the reference genome with HISAT2 [43]. Next, transcripts were assembled and compared using StringTie [44] in combination with GffCompare [45].

We then applied stringent criteria, retaining only transcripts longer than 200 bp with class codes u, x, i, j, or o, and that contained at least one exon. Last, we assessed protein-coding potential using CPC2 [46] and CNCI [71], filtering out any transcripts overlapping with *D. melanogaster* protein sequences. This process identified 14,146 putative lncRNAs in *D. melanogaster*. Further refinement revealed 5246 lncRNAs expressed in at least one sample (Figure 1B).

Additionally, we identified 27,642 mRNAs and 39,051 TEs in the fly genome using RepeatMasker (Figure 1B). Notably, our analysis revealed that a substantial proportion, constituting 40.39% (2119) of lncRNAs, were TE-lncRNAs, characterized by their overlap with TE sequences (Figure 1C). In contrast, only 20.69% of mRNAs were classified as TE-mRNAs which are mRNAs overlapping with transposons (Figure 1C).

### 3.2. Characterization of TE-lncRNAs

**Figure 2:**
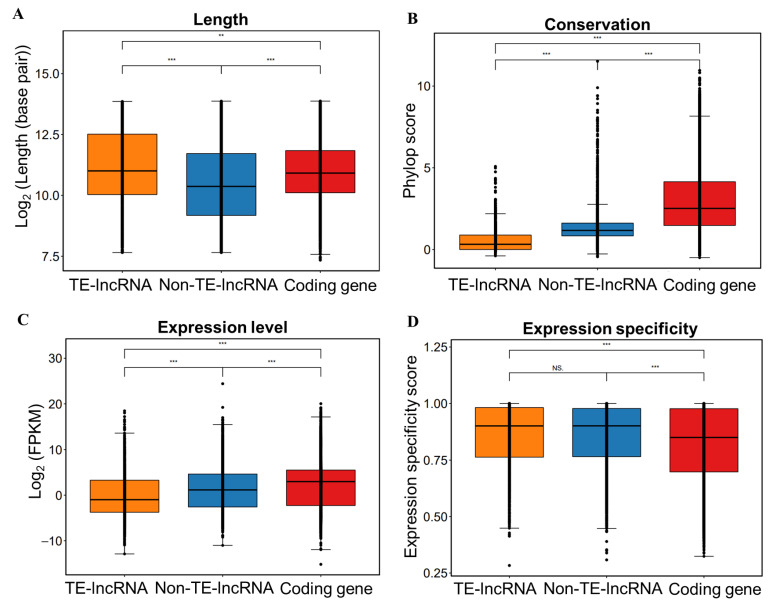
The characteristics of the lncRNAs derived from TEs. (**A**) The length of TE-lncRNAs, Non-TE-lncRNAs, and coding genes. (**B**) Phylop scores among TE-lncRNA, Non-TE-lncRNA, and coding genes sequence. (**C**) The expression levels among TE-lncRNAs, Non-TE-lncRNAs, and coding genes. (**D**) The expression specificity of TE-lncRNAs, Non-TE-lncRNAs, and coding genes. Statistical significance: **: *p* < 0.01; ***: *p* < 0.001; NS.: *p* ≥ 0.05.

We performed a comprehensive comparison of TE-lncRNAs, Non-TE-lncRNAs, and coding genes, focusing on their length, location, conservation, and expression profiles to uncover the distinct characteristics of TE-lncRNAs. Our analysis revealed that TE-lncRNAs have an average length of 12,377.92 base pairs (bp), which is significantly longer than that of Non-TE-lncRNAs (3580.13 bp on average; *t*-test: *p* < 0.001) and coding genes (6959.49 bp on average; *t*-test: *p* < 0.01) (Figure 2A).

Next, we assessed the conservation levels of coding genes, Non-TE-lncRNAs, TE-lncRNAs, and TE insertion sequences using Phastcons and Phylop conservation scores. Notably, coding genes exhibit the highest conservation, followed by Non-TE-lncRNAs and TE-lncRNAs (Figure 2B). Both TE-lncRNAs and TE insertion sequences have a significant proportion of sequences with conservation scores of zero, indicating lower conservation levels (Figures S1 and S2).

Additionally, we analyzed the expression levels and specificity of coding genes, Non-TE-lncRNAs, and TE-lncRNAs across the 15 lncRNA-seq datasets, which includes samples treated with various heavy metals. This allowed us to examine their expression patterns under different conditions. Principal component analysis (PCA) revealed a distinct expression profile associated with TE-lncRNAs in response to heavy metal exposure in comparison to Non-TE-lncRNAs and coding genes (Figure S3). Expression heatmaps showed that most TE-lncRNAs were highly induced by at least one heavy metal (Figure S4). Genes with element-specific expression likely play key roles in cellular responses to heavy metal exposure. The results indicated that coding genes had the highest expression levels, while TE-lncRNAs had the lowest expression levels. TE-lncRNAs presented higher expression specificity than coding genes (Figure 2C,D). However, the difference in specificity between TE-lncRNAs and Non-TE-lncRNAs was not substantial.

### 3.3. Characteristics of TE Insertions in lncRNAs

**Figure 3:**
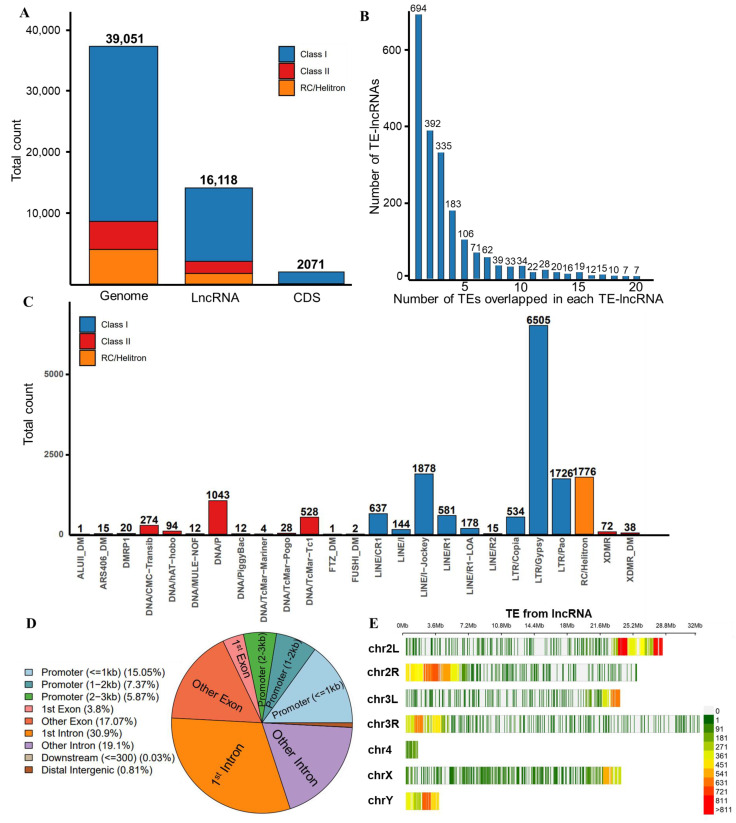
The contribution of TEs to lncRNAs. (**A**) The quantities of various types of TEs that overlap with genome, lncRNA, and CDS sequences. (**B**) Number of TEs overlapping with each TE-lncRNA. (**C**) The quantities of various types of TEs that overlap with lncRNAs. (**D**) Positional preference of TE insertions in lncRNAs. (**E**) Hotspots of TE insertion on chromosomes.

We conducted a comprehensive analysis of the impact of TEs on lncRNAs in *D. melanogaster*. Our findings revealed 16,118 TEs overlapping with lncRNA sequences, averaging 7.6 TEs per lncRNA, compared to just 0.17 TEs per coding gene (Figure 3A). A significant proportion of TE-lncRNAs harbored multiple transposon sequences, with 67.2% containing more than one TE. Some TE-lncRNAs hosted up to 20 or more TEs (Figure 3B). The percentage of TEs inserted in lncRNA is 41.27% (16,118/39,051), while only 5.30% (2071/39,051) are in CDS (Fisher’s exact test: *p* < 0.001). These results indicate that noncoding regions have abundant transposon insertions compared to coding regions.

Class I TEs constitute the majority of TEs associated with lncRNAs (Figure 3A). The Gypsy family has a proportion of 37.32% in the genome, 40.36% in lncRNA, and only 3.04% in CDS (Fisher’s exact test: *p* < 0.001). However, the Pao family has a proportion of 12.26% in the genome, 10.71% in lncRNA, and highly 90.39% in CDS (Fisher’s exact test: *p* < 0.001). Notably, the LTR/Gypsy family within Class I is the most prevalent, comprising 6505 Gypsy elements and accounting for 39% of the total TEs from lncRNAs (Figure 3C and Figure S5A,B). Other significant contributors include LINE/I-Lockey, RC/Helitron, and LTR/Pao.

Our investigation into the length distribution of transposon sequences that overlapped with lncRNAs revealed that they predominantly fell within the range of 0–15,000 bp. Additionally, the coverage rate of transposon insertions within lncRNAs was primarily concentrated within the 0–25% and 75–100% intervals (Figure S7).

We analyzed the spatial distribution of transposon insertions within lncRNAs. The results showed that transposons preferred to insert into the intronic and promoter regions (Figure 3D and Figure S1E). Furthermore, our investigation into the contribution of transposons that overlap with lncRNAs to transcription start sites (TSSs) revealed a predominant involvement of TE-lncRNAs, indicating that transposons may contribute to the formation of essential gene features (Figures S5C,D and S6). Intriguingly, our analysis of transposon insertion hotspots along chromosomes revealed a bias towards chromosome ends in *D. melanogaster* (Figure 3E and Figure S5F).

### 3.4. Epigenetic Regulation Involving TE-lncRNAs 

**Figure 4:**
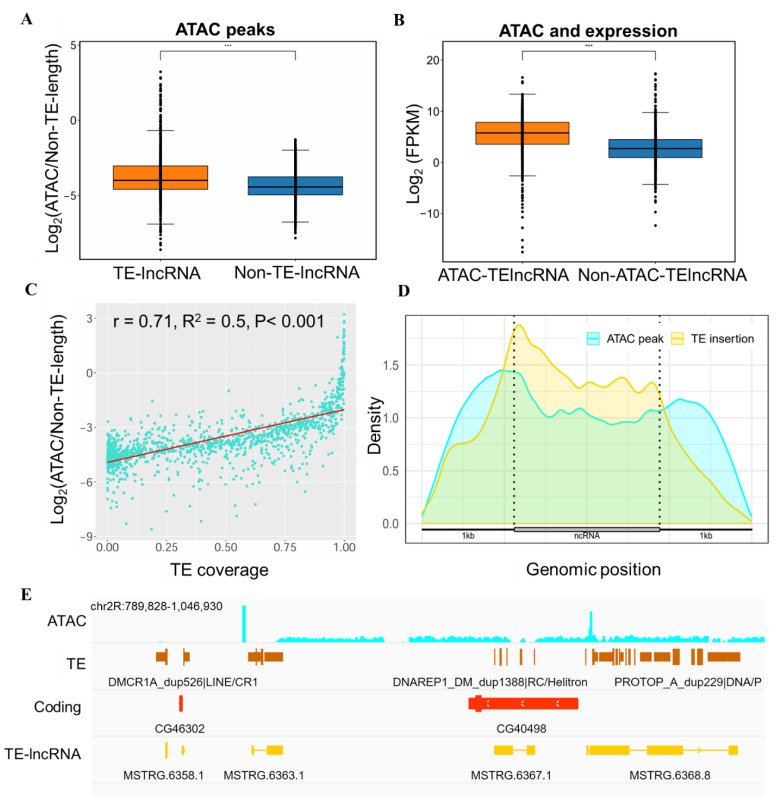
Impact of TE-lncRNAs on epigenetic levels in *Drosophila.* (**A**) ATAC peaks in lncRNAs including TE-lncRNA and Non-TE-lncRNA. (**B**) Expression of ATAC-TE-lncRNA and Non-ATAC-TE-lncRNA. (**C**) The correlation between TE coverage and ATAC peaks. (**D**) The distribution of TE and ATAC peaks in lncRNA. (**E**) IGV view of the ATAC peaks in TE-lncRNA. Statistical significance: ***: *p* < 0.001.

Here, we utilized ATAC-seq data to assess chromatin accessibility across 28 datasets, and identified a total of 372,119 peaks indicative of open chromatin states within lncRNA regions by merging these datasets. We hypothesized that transposon insertions are more prevalent in regions with open chromatin, which facilitates insertion and transcription factor recruitment, while closed and condensed chromatin limits these processes. Additionally, an open chromatin configuration is conducive to the recruitment of transcription factors, thereby initiating the transcriptional process. Our analysis revealed a significant difference in the number of open chromatin peaks between TE-lncRNAs and Non-TE-lncRNAs in *D. melanogaster*. Specifically, 1375 TE-lncRNAs contained 240,354 ATAC peaks, whereas 2774 Non-TE-lncRNAs had only 131,765 ATAC peaks. Remarkably, TE-lncRNAs had twice as many open chromatin regions as Non-TE-lncRNAs. Statistical analysis showed that TE-lncRNAs had significantly more ATAC peaks than Non-TE-lncRNAs (Figure 4A). Additionally, TE-lncRNAs with ATAC peaks exhibited higher expression levels compared to those lacking ATAC peaks (Figure 4B).

We further assessed the transposon coverage length, transposon count, ATAC peak count, transcription factor binding sites (TFBSs), and transposon coverage rate for each lncRNA (Table S6). Correlation analyses among these variables revealed the following notable associations: a positive correlation between transposon quantity and coverage length (Figure S8H), as well as a positive correlation between transposon presence and coverage with the quantity of ATAC peaks in lncRNAs (Figure 4C and Figure S8I). This indicates that a higher transposon content in lncRNAs is associated with an increased ATAC signal intensity. Moreover, we found a positive correlation between the number of ATAC peaks and TFBSs (Figure S8G).

Our examination of transposon and open chromatin state distribution in *Drosophila* lncRNA regions showed striking similarities in the frequency density of ATAC peaks and transposon distribution areas (Figure 4D). Examples from two lncRNAs, *MSTRG.6363.1* and *MSTRG.6368.8*, illustrate the contribution of TEs to ATAC peaks (Figure 4E). These findings indicate that transposon insertion is closely associated with chromatin accessibility, with transposons favoring regions of open chromatin.

### 3.5. Transcriptional Regulation and Co-Expression Analysis for TE-lncRNAs

**Figure 5:**
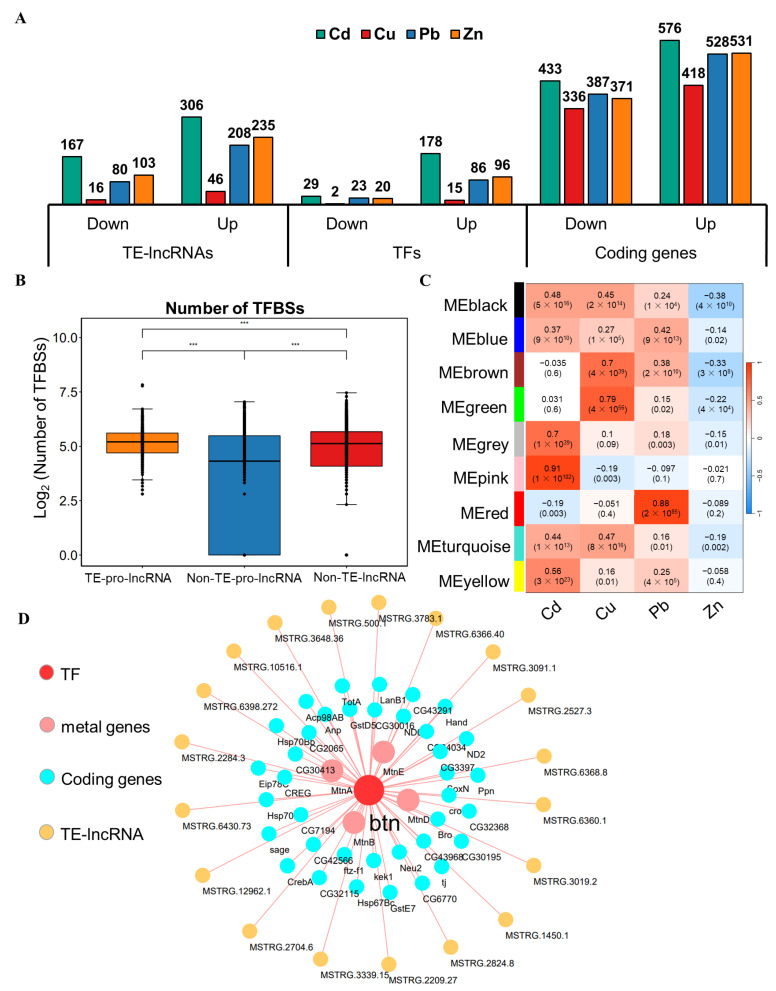
Co-expressed regulatory networks of TE-lncRNAs at the transcriptional level. (**A**) Differentially expressed genes under heavy metal conditions. (**B**) The quantities of TFBSs between TE-lncRNAs and Non-TE-lncRNAs. (**C**) The correlation between gene modules and heavy metal samples; each ME color represents a different expression module. (**D**) The TF regulatory networks mediated by transposons. Statistical significance: ***: *p* < 0.001.

To explore the functional roles of TE-lncRNAs at the transcriptional level, we performed a differential expression analysis to identify the TE-lncRNAs, transcription factors, and protein-coding genes involved in responses to heavy metal exposure (Figure 5A), and scanned the TFBSs for lncRNAs; the most enriched TFBSs are listed in Supplementary Table S7. We identified 3580 coding genes, 1161 TE-lncRNAs, and 49 transcription factors that were significantly induced. The number of TE-lncRNAs either upregulated or downregulated by exposure to the four heavy metals varied greatly compared to coding genes (Figure 5A).

We assessed the abundance of TFBSs within the promoter regions of lncRNAs by categorizing TE-lncRNAs into two groups: those with transposons in their promoters (TE-pro-lncRNAs) and those without (Non-TE-pro-lncRNAs). The results showed that TE-lncRNAs with transposon promoters had a higher density of TFBSs compared to those without transposon promoters and Non-TE-lncRNAs (Figure 5B). Additionally, we identified 516 distinct TE-lncRNAs where transposons contributed TFBSs within the lncRNA sequences (Figure S9A). Among these, 38 TE-lncRNAs exhibited heightened expression specifically under heavy metal treatment in S2 cells, indicating some degree of heavy metal specificity (Figure S9B). The main contributors to these TFBS-containing TE-lncRNAs were the LTR/Gypsy, LTR/Pao, and LINE/I-Jockey transposon families (Figure S9B).

We next validated the roles of TE-lncRNAs in regulatory networking mediated by transposons. By merging expression matrices from 271 samples encompassing TE-lncRNAs, transcription factors, and differentially expressed protein-coding genes, we constructed a comprehensive co-expression network (Figure S9C). Within this network, we identified known heavy metal responsive genes such as *MtnA*, *MtnB*, *MtnD*, and *MtnE*, which were clustered in a grey subnetwork module highly correlated with Cd stress samples (Figure 5C). Further analysis revealed the distribution of TFBSs within TE-lncRNAs and chromatin-accessible regions within the grey module, leading to the delineation of a regulatory network centered around the *btn* transcription factor (Figure 5D). For instance, *btn* exhibited binding sites on 19 TE-lncRNAs, including the promoter region of the TE-lncRNA, which displayed an open chromatin peak possibly facilitated by the transposon insertion, thereby providing a binding site for *btn* on this TE-lncRNA. This transposon-mediated formation of a transcriptional regulatory network contributes to the regulatory functions (Figure 5D).

**Figure 6:**
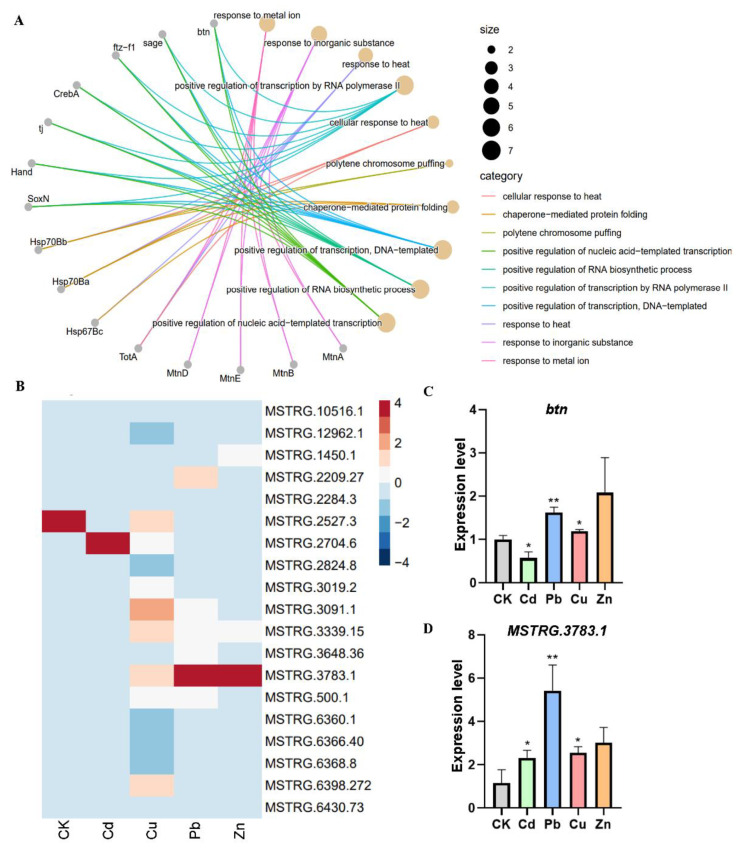
Validation of genes expressed in response to heavy metal stress in *Drosophila* S2 cells. (**A**) GO enrichment analysis of coding genes from those regulatory networks correlated with TE-lncRNAs; grey indicates genes, yellow indicates functional descriptions, and the line between grey circles and yellow circles indicates relations between genes and functions. (**B**) Expression heatmap of TE-*lncRNAs* from the *btn* network. (**C**) Expression validation of *btn* using quantitative PCR (qPCR). (**D**) Expression validation of *MSTRG.3783.1* using qPCR. Statistical significance: *: *p* < 0.05; **: *p* < 0.01.

We performed a further functional enrichment analysis of the protein-coding genes within the network. The analysis indeed revealed enrichment in functions associated with heavy metal response, such as response to metal ion, chaperone-mediated protein folding, and positive regulation of transcription (Figure 6A). Thus, in the transposon-mediated *btn* regulatory network, transposons may furnish binding sites for TE-lncRNAs, enabling the recruitment of transcription factors for transcriptional regulation related to heavy metal stress response. An examination of the TE-lncRNA expression within the co-expression regulatory network heatmap highlighted the highly induced expression level of *MSTRG.3783.1* (Figure 6B). Subsequent quantitative expression measurement validated the specific expression patterns of both *btn* and *MSTRG.3783.1* under heavy metal stress conditions (Figure 6C,D).

### 3.6. Post-Transcriptional Regulation of TE-lncRNAs

To investigate the association between TEs and m^6^A modification sites in lncRNAs, we performed a comparative analysis of m^6^A sites in TE-lncRNAs versus Non-TE-lncRNAs. We observed no significant difference in m^6^A abundance between the two groups (Figure S10A). However, we identified 99 lncRNAs with m^6^A modifications linked to TEs, with 16 of these showing elevated expression in heavy metal-treated S2 cells, suggesting a specific response to heavy metal stress (Figure S10B). Notably, the LTR/Pao and LTR/Gypsy transposon families were significant contributors to the m^6^A-modified TE-lncRNAs (Figure S10B).

**Figure 7:**
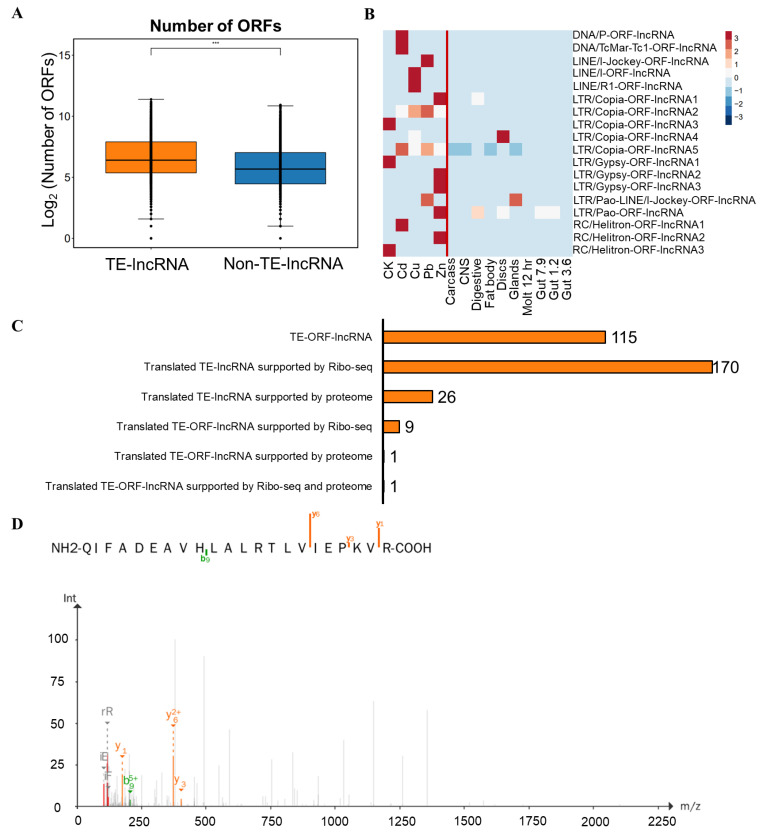
Effects of TE-lncRNAs on post-translational regulation in *Drosophila*. (**A**) Number of ORFs in TE-lncRNAs and Non-TE-lncRNAs. (**B**) Expression level of TE-ORF-lncRNAs in different samples. (**C**) Statistics of translated TE-lncRNAs. (**D**) Peptides encoded by TE-lncRNAs. Statistical significance: ***: *p* < 0.001.

We also annotated open reading frames (ORFs) within lncRNAs and found a higher prevalence of candidate ORFs in TE-lncRNAs compared to Non-TE-lncRNAs, indicating that transposons may contribute to ORF formation within lncRNAs (Figure 7A). Further analysis identified 115 lncRNAs with TE-derived ORFs (TE-ORF-lncRNAs). Among these, 18 TE-ORF-lncRNAs showed increased expression following heavy metal treatment in S2 cells, with significant contributions from the LTR/Copia and RC/Helitron transposon families (Figure 7B). Additionally, Ribo-seq data revealed 170 translated TE-lncRNAs and nine translated TE-ORF-lncRNAs, while proteomics data identified 26 translated TE-lncRNAs and one translated TE-ORF-lncRNA (Figure 7C). Notably, the lncRNA *MSTRG.9753.7*, which contains an ORF derived from a transposon, was confirmed to be translated, while the peptide encoded by *MSTRG.9753.7* was validated using mass spectrometry (Figure 7D).

## 4. Discussion

In this study, we comprehensively identified and functionally characterized a special type of lncRNA, known as TE-lncRNA, based on 256 public *Drosophila* RNA-seq datasets and 15 lncRNA-seq datasets obtained within our laboratory. We identified 5246 lncRNAs, from which 2119 TE-lncRNAs were further identified and overlapped with transposon sequences in the *Drosophila* genome. We here identified many more lncRNAs than those (3085 lncRNAs) in a previous study [40], maybe because more lncRNA-seq transcriptome datasets are included, and a new pipeline and tools were used (see Methods). Previous research has demonstrated that TEs contribute to the origin, diversification, and regulation of lncRNAs in species such as humans, mice, zebrafish, chimpanzees, gorillas, and rhesus [32,33,34]. Our study highlighted the widespread contribution of TEs to lncRNAs in different species as well as in the model insect species, *D. melanogaster*.

Several recent studies have demonstrated the important functional roles of lncRNAs in *Drosophila*. Remarkably, the expression of the aal1 lncRNA in *Drosophila* boosts fly lifespan [72], and the dysfunction of lncRNA:CR43306 contributes to testicular aging [73]. For development and stress, bsAS, an antisense lncRNA, plays an essential role for correct wing development [74]; lncRNA CR40469, in trans responds to damage in the wing imaginal disk [75], and lncRNA NEAT1 is dramatically upregulated in stressed neurons [76]. LncRNAs are also involved in the regulation of obesity, immunity, and metabolisms in *Drosophila* [77]. For example, lncRNA-IRAR mediates the regulation of insulin receptor transcripts [78], and lincRNA-IBIN connects immunity and metabolism [79]. In addition, lncRNA VINR activates a non-canonical antimicrobial defense pathway in response to the VSR of *Drosophila* C virus [80]; CifA and CifB proteins alter lncRNA to establish a paternal-effect embryonic lethality [81]. From molecular aspects, roX lncRNAs are essential components of the chromatin modifying Dosage Compensation Complex (DCC) in *Drosophila* [82]. Meanwhile, lncRNAs play major roles in evolution by controlling transposable element activities, Y chromosome gene expression, and sperm construction [19]. Interestingly, some lncRNAs in *Drosophila* even could encode micropeptides [83].

Our analysis revealed that the LTR/Gypsy family represents the largest proportion (39%) of TEs within TE-lncRNAs in *D. melanogaster*, compared to its relatively minor presence (3%) in coding sequences. This suggests a preferential insertion of the LTR/Gypsy family into TE-lncRNAs. This finding is in line with previous studies across various species, where the LTR/Gypsy family is consistently the most abundant transposon family within lncRNAs [31,84,85,86]. In contrast, the ERV family is the most enriched for TE-lincRNAs in humans, while Alu elements predominate in mice TE-lincRNAs [32]. Among plants, different TE families are the primary contributors to TE-lincRNAs, namely Helitron in *Arabidopsis*, MITEs in rice, and Gypsy in maize [84]. These results suggest a potential role for these retained transposons in contributing to lncRNAs.

TE-lncRNAs differ from other gene types in several respects, including length, location, conservation, and expression levels. Notably, TE-lncRNAs are longer than Non-TE-lncRNAs. This finding suggests that transposon insertions contribute to the elongation of TE-lncRNAs, potentially increasing their resilience to transposon insertion. TE-lncRNAs also overlap with promoter and transcription start site (TSS) regions, suggesting they may function as regulatory elements. TE-lncRNAs are less conserved than other lncRNAs and coding genes. This variation in conservation among different gene types likely reflects the dynamic nature of transposon activity, leading to sequence variations and genomic instability.

Despite these potential functions, TE-lncRNAs are relatively poorly conserved, reflecting their origins from transposon insertions. They exhibit high specificity but low overall expression across various species, including humans, mice, zebrafish, and several plants [32,33,84,87,88]. TEs are often tightly regulated due to their potential disruptive impact on the genome and gene regulatory elements [27]. The piRNA system can control TE mobility by both transcriptional gene silencing and post-transcriptional gene silencing [89]. Thus, although the chromatin is accessible, active suppression mechanisms still could prevent TE-lncRNAs from being expressed at high levels.

We conducted a comparative analysis of the positioning of TEs genome-wide and in all TE-lncRNAs in *D. melanogaster*. A higher occurrence of TE-lncRNAs at transposon hot sites across the genome was observed. Notably, the majority of transposon insertions from TE-lncRNAs were concentrated within intronic regions, with a subset contributing to promoter and TSSs regions, which is consistent with previous findings [32]. This preference suggests that the intronic region may exhibit greater tolerance to transposon insertion, while insertion in promoter regions could influence the regulation of nearby lncRNAs. This finding underscores the functional potential of transposon sequences, particularly when inserted into regulatory regions associated with biological activities. Of particular interest are several hotspots of transposon insertions on chromosomes, which are notably concentrated close to either end of some chromosomes or even within subtelomeric regions. Subtelomeric regions are also transcribed into coding and noncoding RNAs, such as ARRET, αARRET, subTERRA, and TERRA [90]. The establishment of open DNA replication-prone structures in subtelomeric regions could be mediated by these lncRNAs and even some TE-lncRNAs.

Epigenetic analysis revealed a significant positive correlation between the abundance of TEs in lncRNA regions and open chromatin regions. This supports the idea that transposons integrate into accessible chromatin, which may facilitate gene expression regulation by providing additional TFBSs. Our study also found that TE-lncRNAs with TEs in their promoter regions had a higher abundance of TFBSs compared to Non-TE-lncRNAs. This association suggests that transposons within lncRNAs may either provide functional TFBSs or facilitate their creation, facilitating transcription factor recruitment and enhancing the transcriptional regulatory functions of lncRNAs.

We used a heavy metal-induced *Drosophila* S2 cell model to explore the regulatory roles of transposon-mediated networks under stress. Differential expression analysis encompassing TE-lncRNAs, transcription factors, and coding genes revealed several well-known heavy metal responsive genes such as *MtnA, MtnB*, *MtnD*, and *MtnE* in a subnetwork module [91,92,93]. A regulatory network centered around transcription factor *btn* was delineated, in which *btn* exhibited binding sites on 19 TE-lncRNAs, including the promoter region of TE-lncRNA. Through meticulous screening for TE-lncRNAs exhibiting high correlation, this study also identified *MSTRG.3783.1* as a heavy metal-responsive TE-lncRNA. However, the specific functional role of this TE-lncRNA awaits further investigation. In terms of biological functions, *btn* is also the *Drosophila* homologue of MEOX2 in primary afferent nociceptor neurons that is proposed for the maintenance of a transcriptional program required for proper perception of acute and inflammatory noxious stimuli [94]. The *btn* contributes to cuticle pigmentation, and one biological process function for *btn* is “response to stimulus” such as noxious heat stimuli [94]. Thus, although no reports show a direct role of *btn* in response to metal, *btn* is implicated for getting involved in regulating heavy metal response and its specific regulatory role awaits further experimental validation.

At the post-transcriptional level, we examined m^6^A modification sites within lncRNAs and found no significant difference between TE-lncRNAs and Non-TE-lncRNAs, indicating a weak connection between TEs and m^6^A modification. However, translational analysis showed that TE-lncRNAs have more ORFs compared to Non-TE-lncRNAs, likely due to their longer length or the insertion of transposons with additional ORFs. Ribo-seq and proteomics data identified translated TE-lncRNAs, providing evidence that TEs contribute to the translation of lncRNAs and the encoding of short peptides. This suggests that TEs play a significant role in expanding the functional repertoire of lncRNAs.

The analysis findings regarding transposons providing TFBSs and ORF elements for lncRNAs corroborate the RIDL hypothesis, which posits that transposon insertion into lncRNAs can function akin to domains found in proteins [95]. Through a multi-omics approach, transposons are shown to facilitate the genesis of lncRNAs and exert regulatory influences by furnishing diverse functional elements for their functionality. By elucidating the presence and impacts of transposons at various omics tiers, we offered a new insight for exploring the intricate relationship between transposons and lncRNAs. One shortcoming might be the complexity of distinguishing the functional contributions of transposon-derived sequences from those of native genomic elements within lncRNAs. Additionally, the multi-omics approach, while comprehensive, could be influenced by dataset biases or technical limitations in detecting transposon activity at different omics levels. To address these challenges, more experiments on transposon activity and regulatory impact may be needed in the future.

## 5. Conclusions

This study offers a comprehensive identification and analysis of TE-lncRNAs in *D. melanogaster*, elucidating their distinctive features and regulatory roles. A multi-omics integrated analysis approach helped unravel the complex regulatory networks of TE-lncRNAs. These findings collectively highlight the regulatory and functional significance of transposon-mediated mechanisms for shaping the landscape of lncRNA biology, opening avenues for future research on TE-lncRNAs.

## Data Availability

The data presented in the study are available in the Supplementary Materials.

## Supplementary Materials

The following supporting information can be downloaded at: https://www.mdpi.com/article/10.3390/insects15120950/s1. Supplementary Table S1. RNA-seq samples. Table S2. Sample information of RNA-seq. Table S3. Sample information of ATAC -seq. Table S4. Sample information of MeRIP-seq. Table S5. Sample information of Ribo-seq. Table S6. TEs, TFBS, ATAC information in lncRNAs. Table S7. TFBS motifs in lncRNAs. Figure S1. Phastcons conservation assessment of coding genes, Non-TE-lncRNA, TE-lncRNA, and TE insertion sequences. (A) Phastcons score of coding genes. (B) Phastcons score of Non-TE-lncRNAs. (C) Phastcons score of TE-lncRNAs. (D) Phastcons score of TE insertions. Figure S2. Phylop conservation assessment of coding genes, Non-TE-lncRNA, TE-lncRNA, and TE insertion. (A) Phylop score of coding genes. (B) Phylop score of Non-TE-lncRNAs. (C) Phylop score of TE-lncRNAs. (D) Phylop score of TE insertions. Figure S3. Principal component analysis of coding genes, Non-TE-lncRNAs, and TE-lncRNAs in heavy metal samples. (A) PCA of coding genes. (B) PCA of Non-TE-lncRNAs. (C) PCA of TE-lncRNAs. Figure S4. Expression level of coding genes, Non-TE-lncRNAs, and TE-lncRNAs under heavy metal conditions. (A) Heatmap of expression for coding genes. (B) Heatmap of expression for Non-TE-lncRNAs. (C) Heatmap of expression for TE-lncRNAs. Figure S5. The contribution assessment of TEs in *D. melanogaster*. (A) Number of transposons from genome, CDS, and lncRNA. (B) Proportions of transposons from TE-lncRNA. (C) The TE insertions from lncRNA frequency near TSSs. (D) TEs from lncRNA insert into transcription factor binding loci relative to TSSs. (E) The gene feature of TE insertions from lncRNA. (F) The hotspot of TE insertions on the genome. Figure S6. Positions of TE insertions in the genome. (AB) The gene feature of TE insertions from the genome. (C) The TE insertions from genome frequency near TSSs. (D) TEs from genome insertion into transcription factor binding loci relative to TSSs. Figure S7. Length of TE insertions. (A) Length of TE derived for lncRNA. (B) TE insertion coverage in lncRNA. Figure S8. Correlations among TE number, TE coverage, TE coverage length, TFBSs, and ATAC peaks. (A) Correlations between TE coverage and TE number. (B) Correlations between TFBSs and TE number. (C) Correlations between TE coverage and TE coverage length. (D) Correlations between TFBSs and TE coverage length. (E) Correlations between ATAC peaks and TE coverage. (F) Correlations between TFBSs and TE coverage. (G) Correlations between TFBSs and ATAC peaks. (H) Correlations between TE coverage length and TE number. (I) Correlations between ATAC peaks and TE number. Figure S9. The transcriptional analysis of TE-lncRNA in *D. melanogaster*. (A) Number of TFBSs on TE-promoter-lncRNA. (B) Highly expressed TE-TF-lncRNAs under heavy metal conditions. (C) Co-expression network of heavy metal network. Figure S10. Post-transcriptional analysis of TE-lncRNA in *D. melanogaster*. (A) Comparison of m^6^A peaks between Non-TE-lncRNA and TE-lncRNA. (B) Highly expressed TE-m^6^A-lncRNAs under heavy metal conditions. References [39,41] are cited in the supplementary materials.
