# Supplementary material for: Transposable Elements Contribute to the Regulation of Long Noncoding RNAs in Drosophila melanogaster
Source: Insects. 2024 Nov 30;15(12):950. doi: 10.3390/insects15120950 (PMC11678190; doi:10.3390/insects15120950)
Supplement: Supplementary file 1 [file insects-15-00950-s001.zip › Figure_S6.pdf]

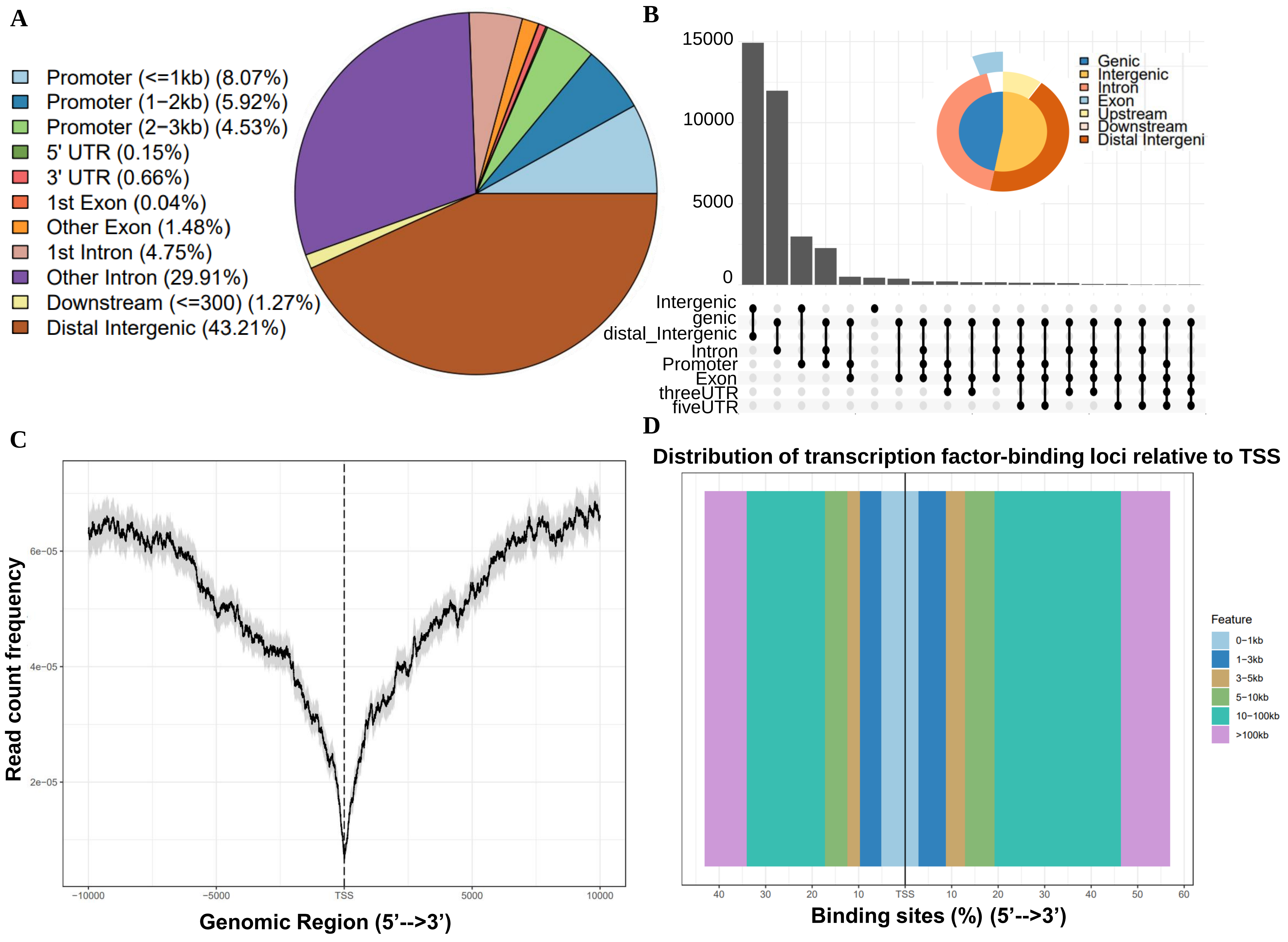

A
Promoter (<=1kb) (8.07%)
Promoter (1−2kb) (5.92%)
Promoter (2−3kb) (4.53%)
5' UTR (0.15%)
3' UTR (0.66%)
1st Exon (0.04%)
Other Exon (1.48%)
1st Intron (4.75%)
Other Intron (29.91%)
Downstream (<=300) (1.27%)
Distal Intergenic (43.21%)
B
15000
10000
5000
0
Genic
Intergenic
Intron
Exon
Upstream
Downstream
Distal Intergeni
Intergenic
genic
distal_Intergenic
Intron
Promoter
Exon
threeUTR
fiveUTR
C
Read count frequency
6e−05
4e−05
2e−05
−10000
−5000
TSS
5000
10000
Genomic Region (5'-->3')
D
Distribution of transcription factor-binding loci relative to TSS
Feature
0−1kb
1−3kb
3−5kb
5−10kb
10−100kb
>100kb
40
30
20
10
TSS
10
20
30
40
50
60
Binding sites (%) (5'-->3')
