# Supplementary figures and images for: Transposable Elements Contribute to the Regulation of Long Noncoding RNAs in Drosophila melanogaster
Source: Insects. 2024 Nov 30;15(12):950. doi: 10.3390/insects15120950 (PMC11678190; doi:10.3390/insects15120950)

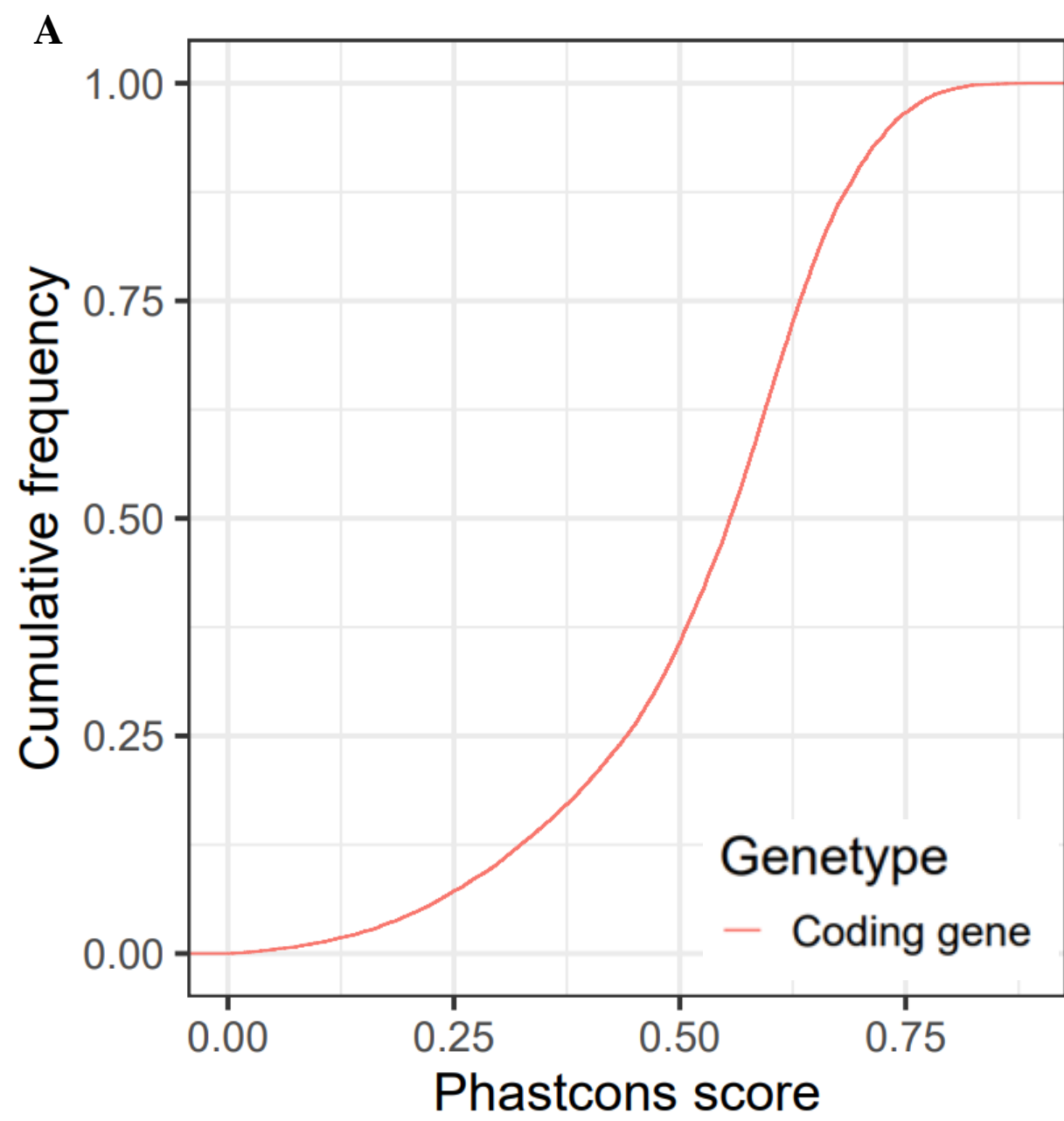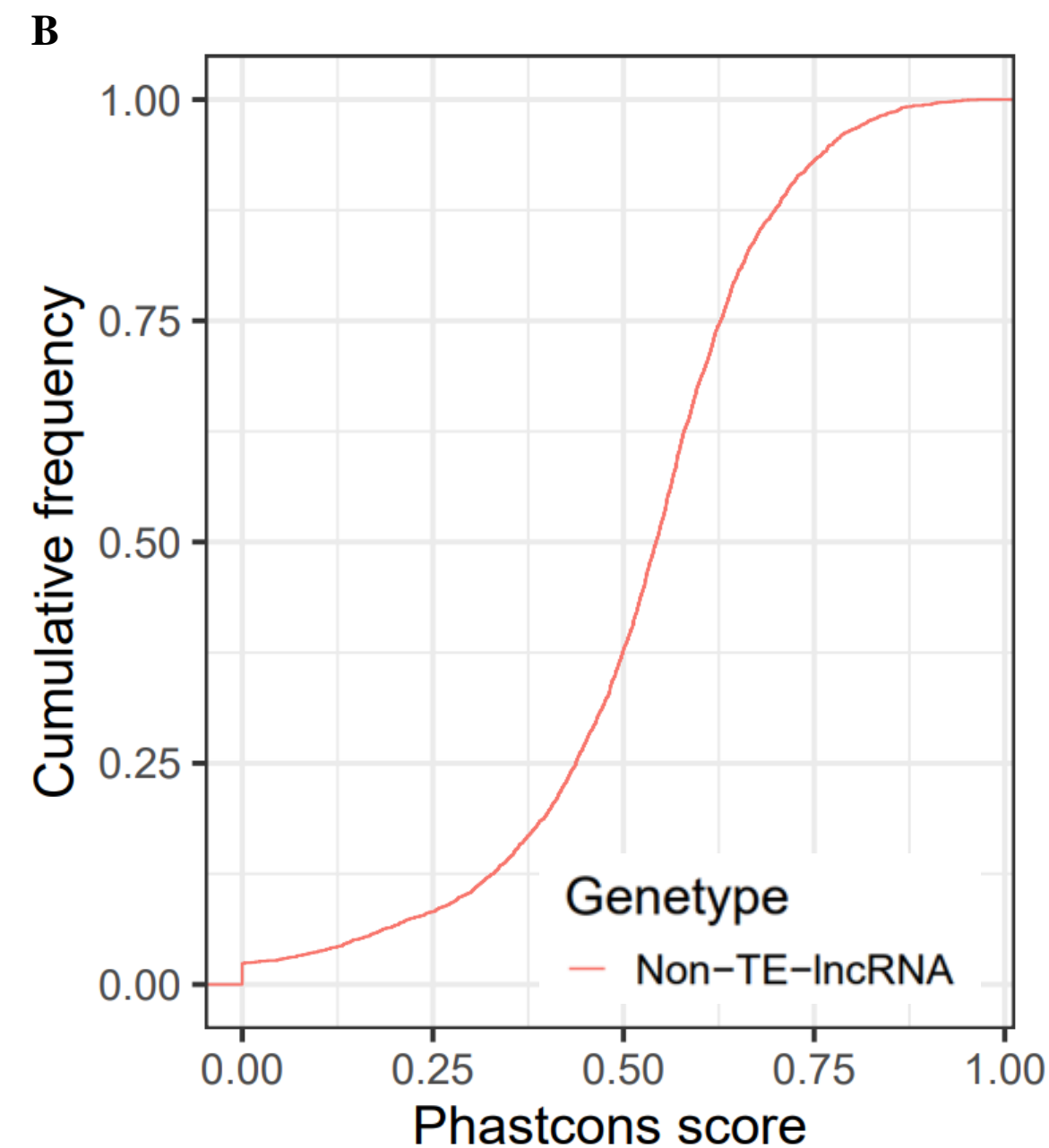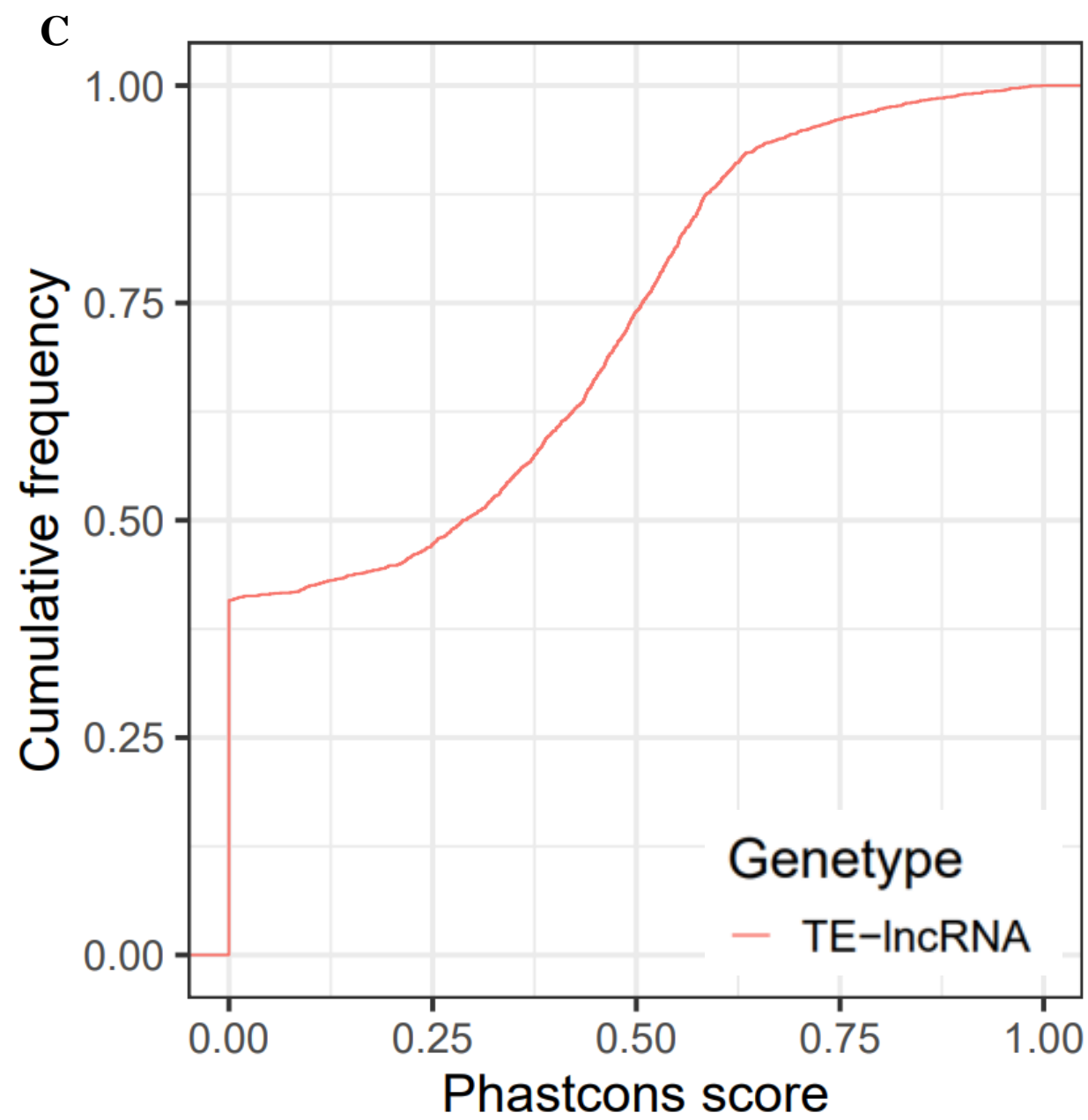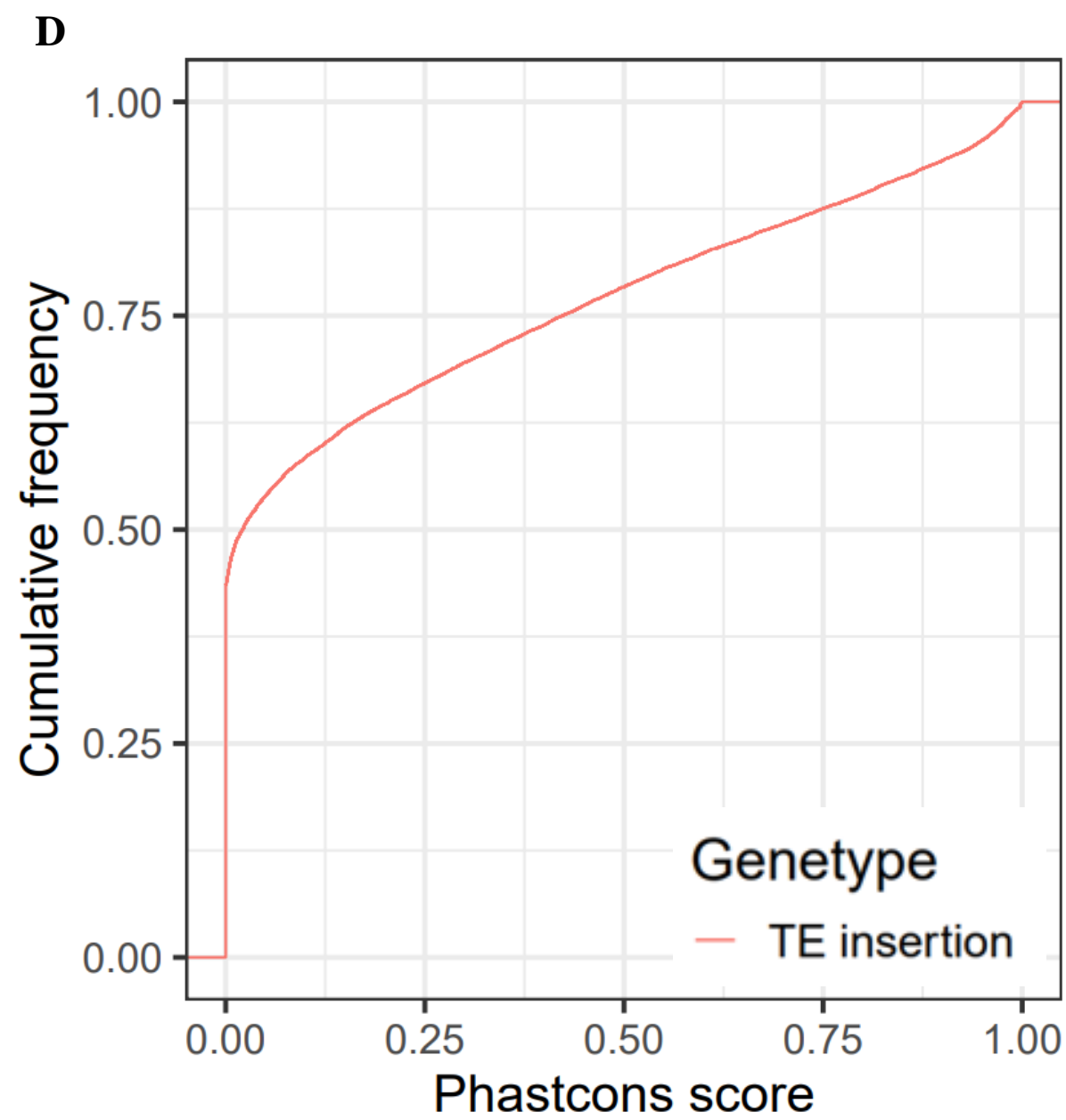

Supplement: Supplementary file 1 [file insects-15-00950-s001.zip › Figure_S1.pdf]

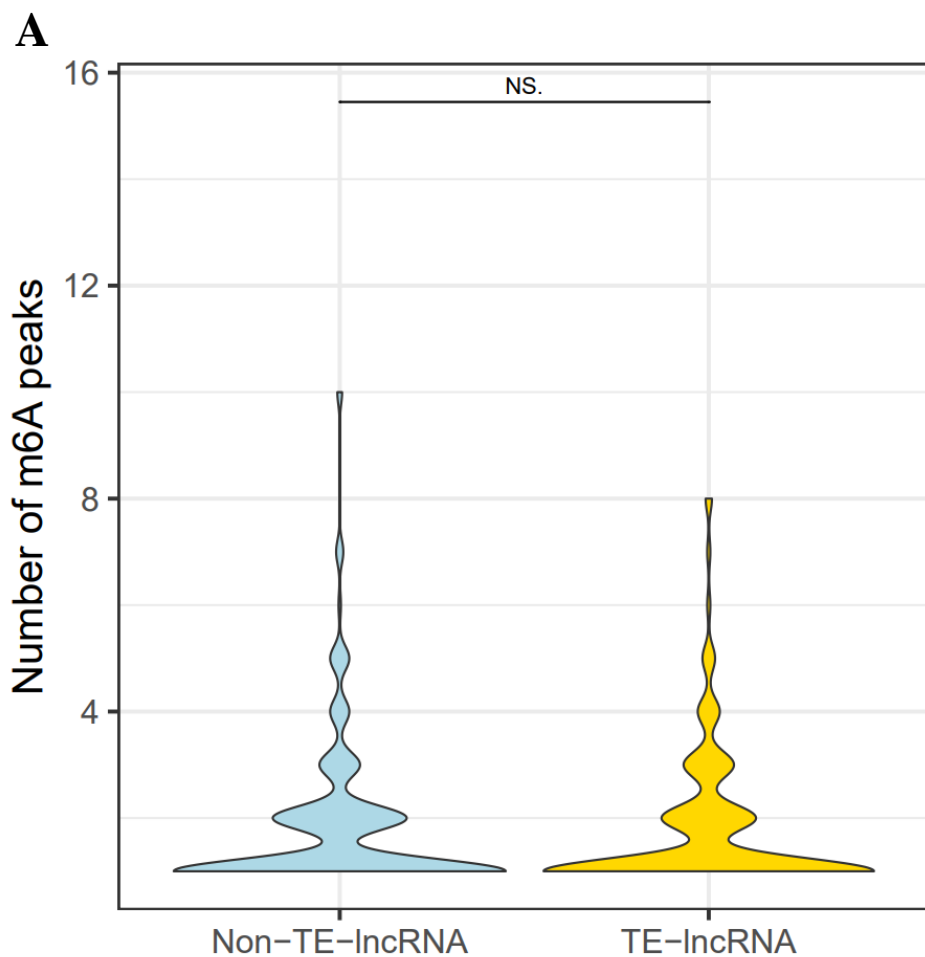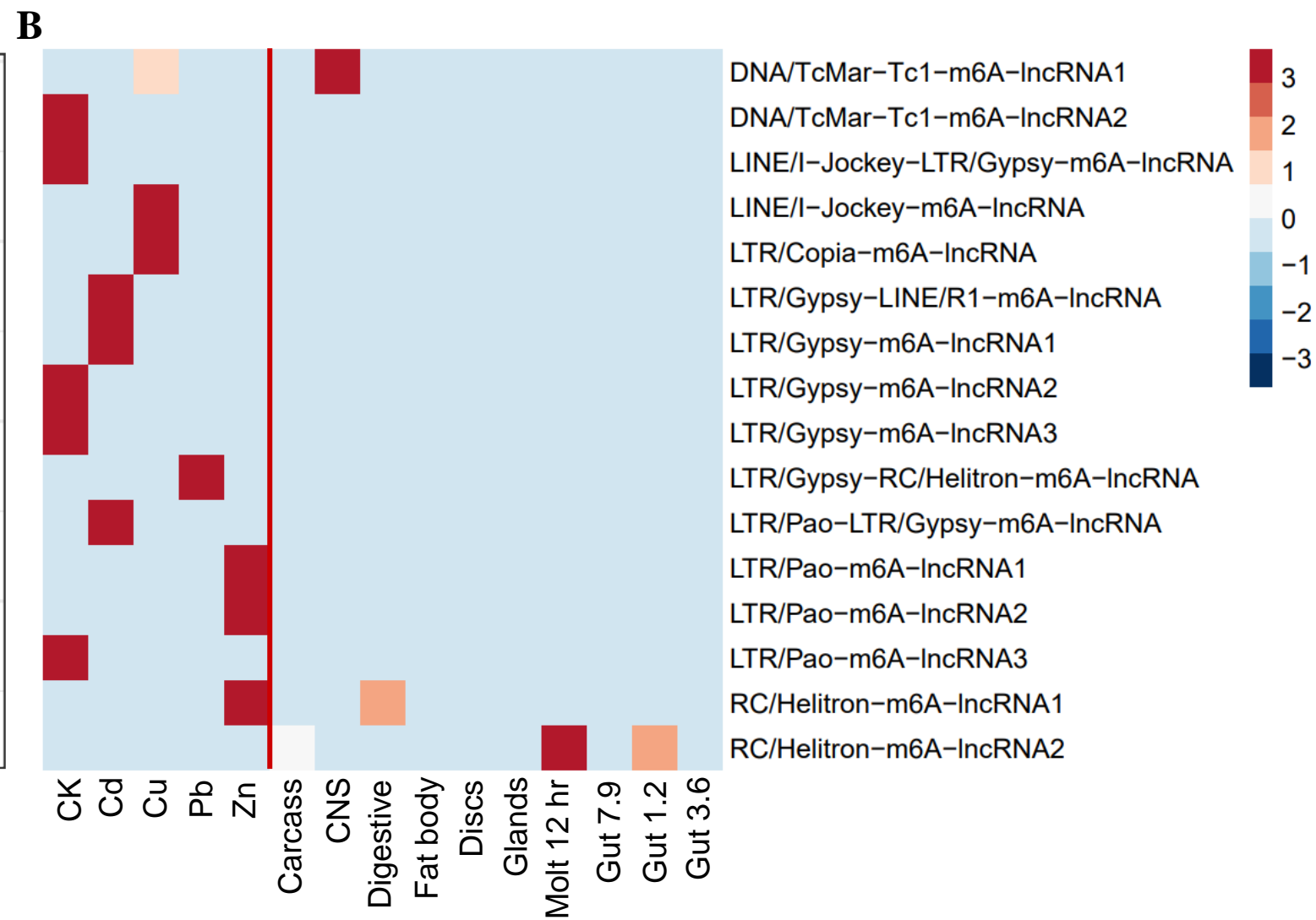

Supplement: Supplementary file 1 [file insects-15-00950-s001.zip › Figure_S10.pdf]

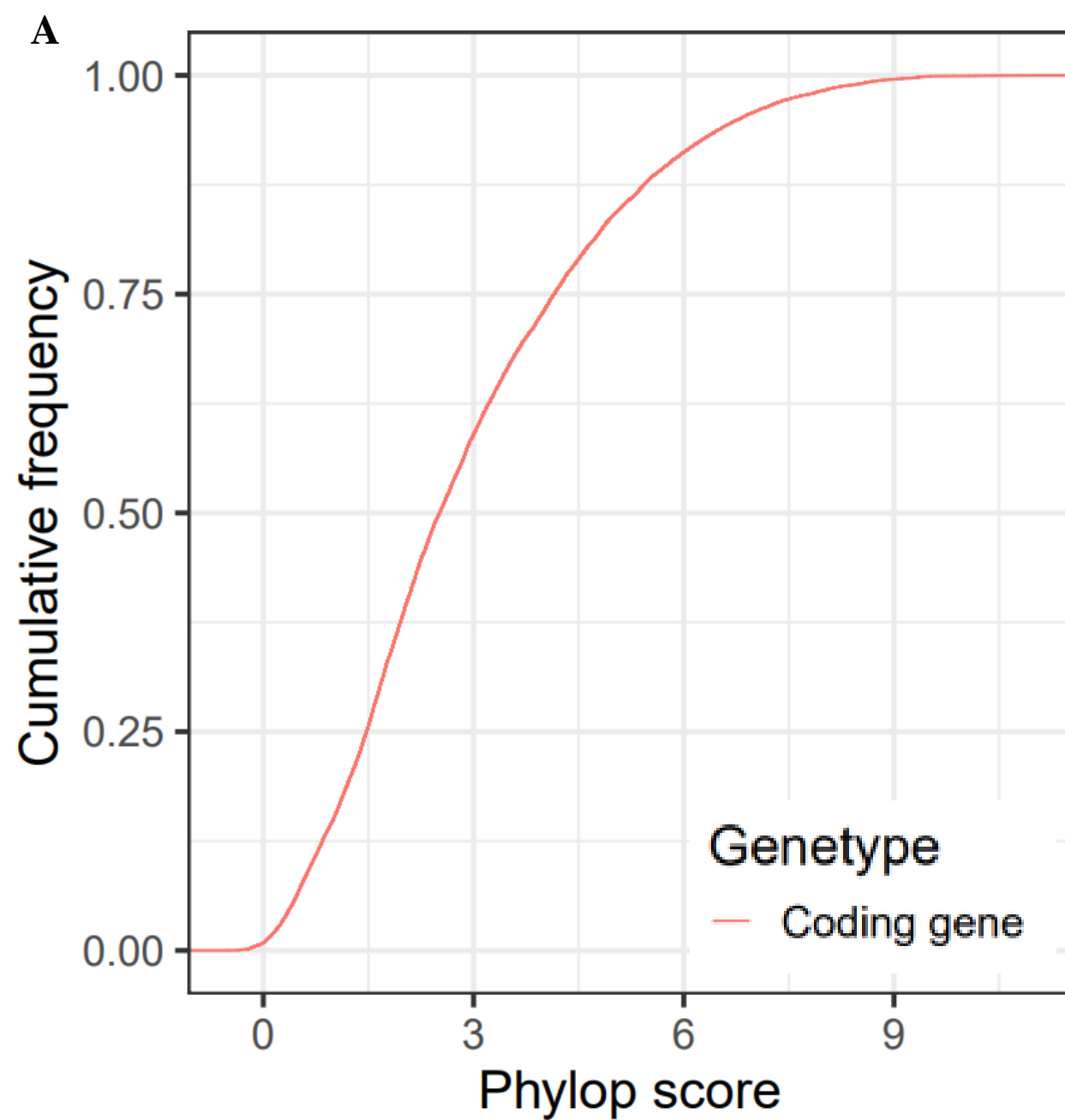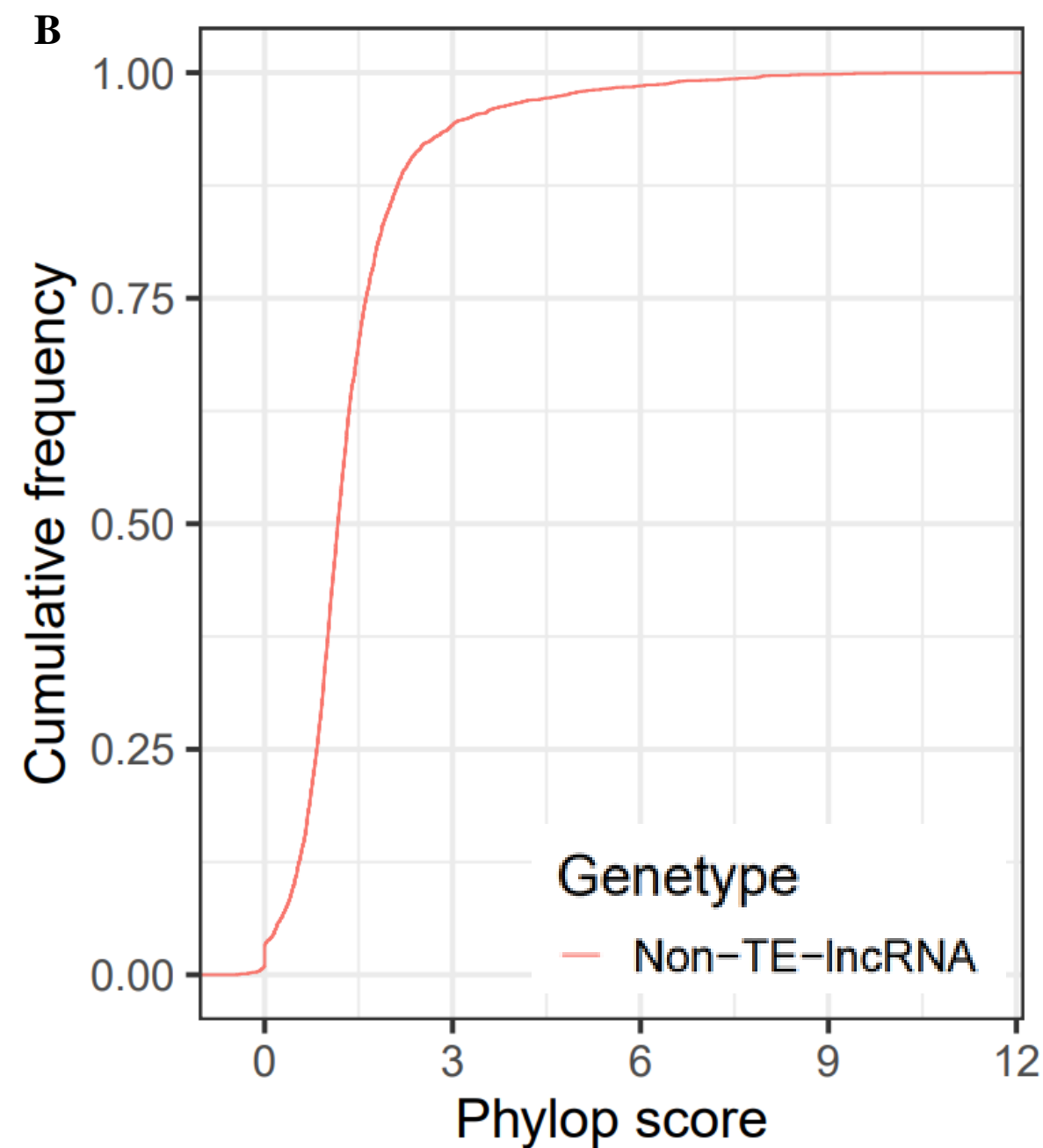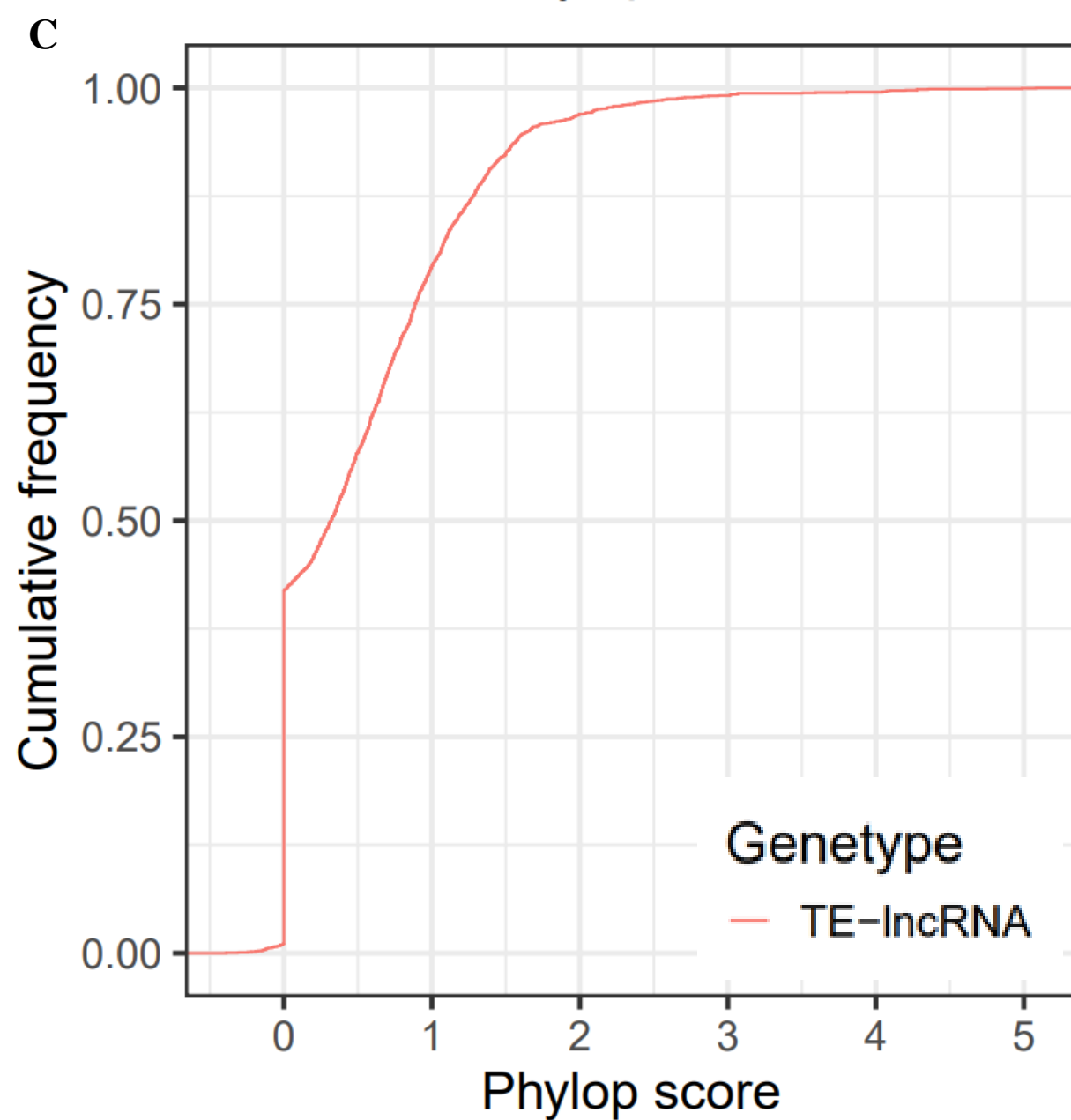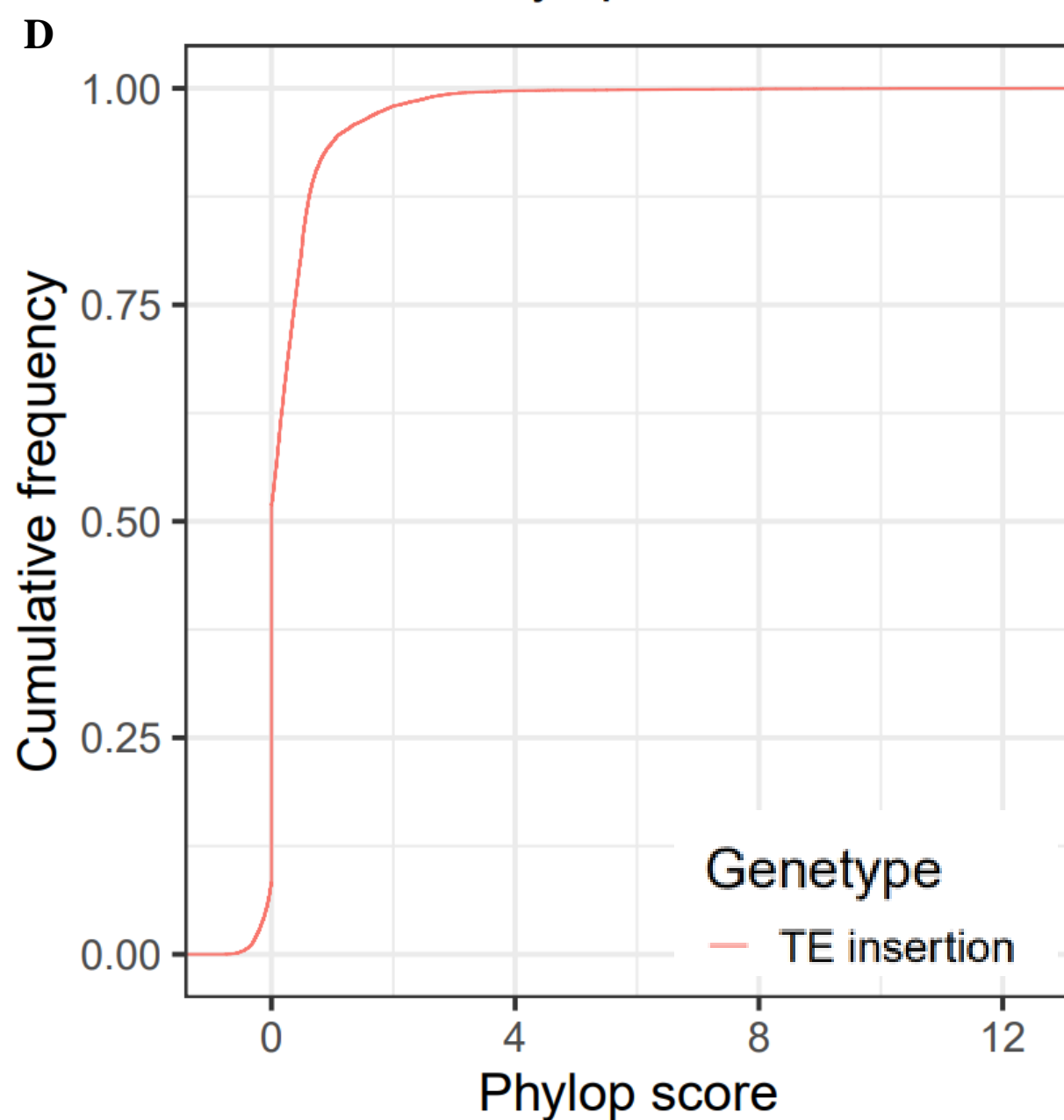

Supplement: Supplementary file 1 [file insects-15-00950-s001.zip › Figure_S2.pdf]

**A****Coding**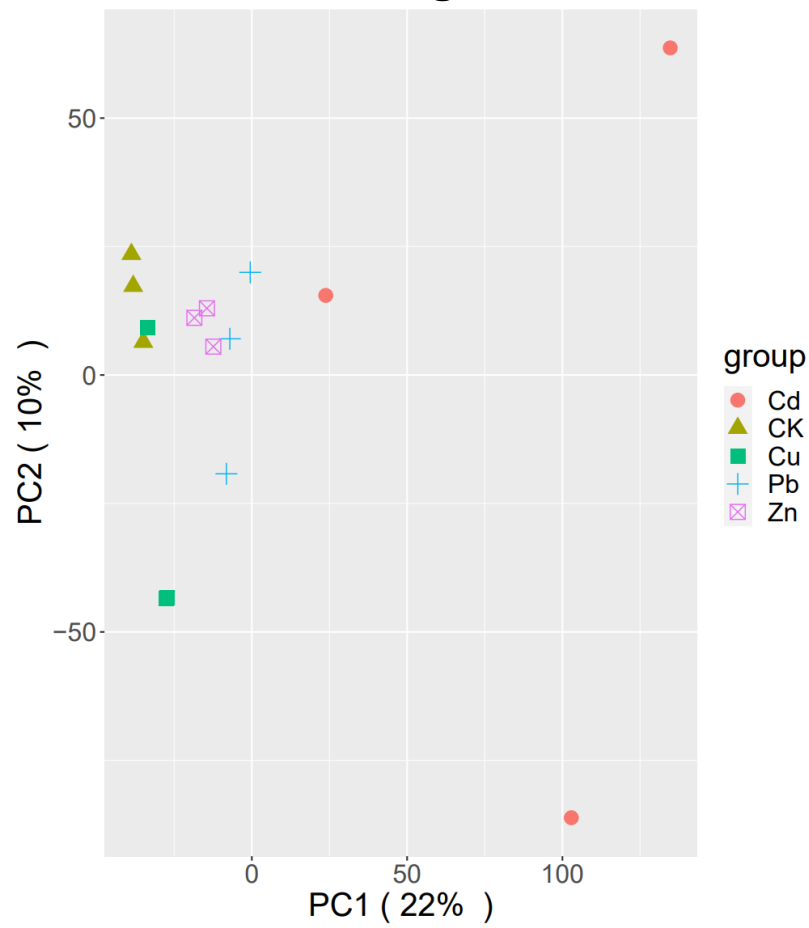**B****Non-TE-IncRNA**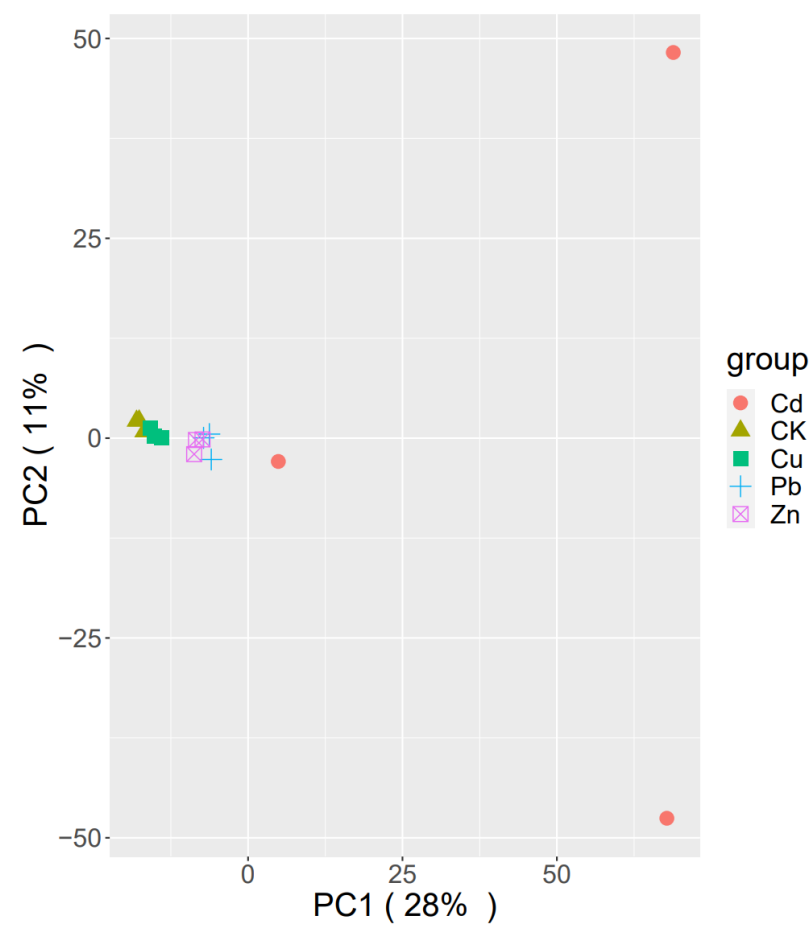**C****TE-IncRNA**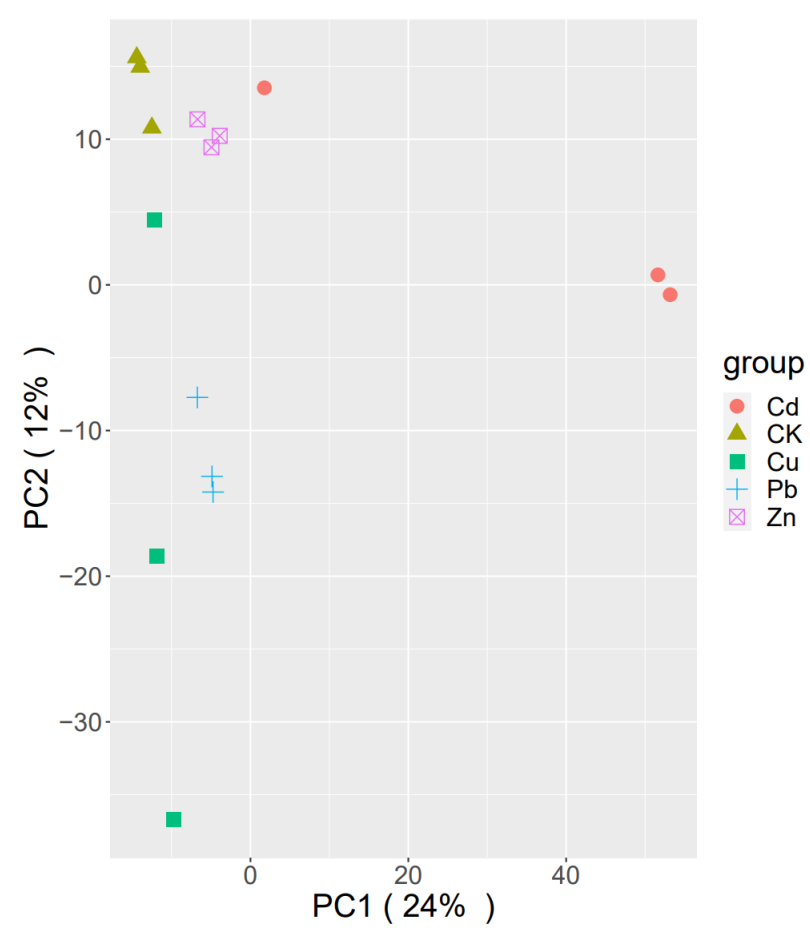

Supplement: Supplementary file 1 [file insects-15-00950-s001.zip › Figure_S3.pdf]

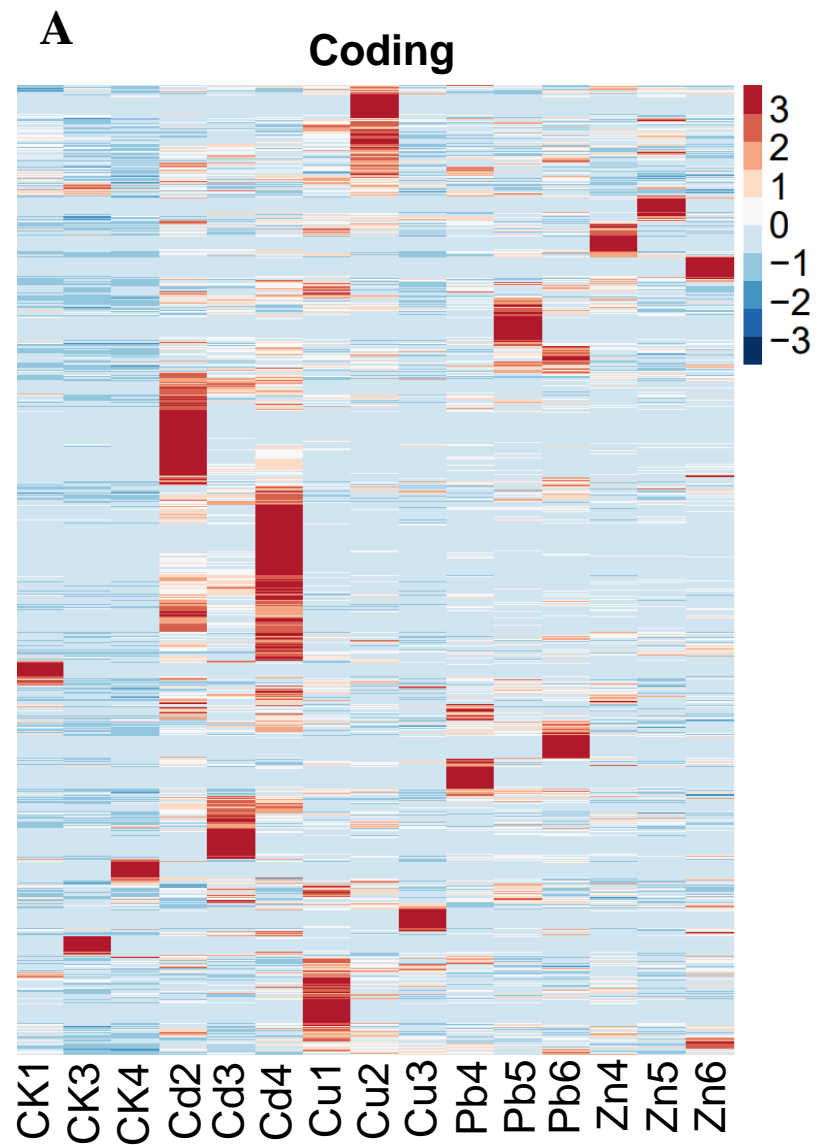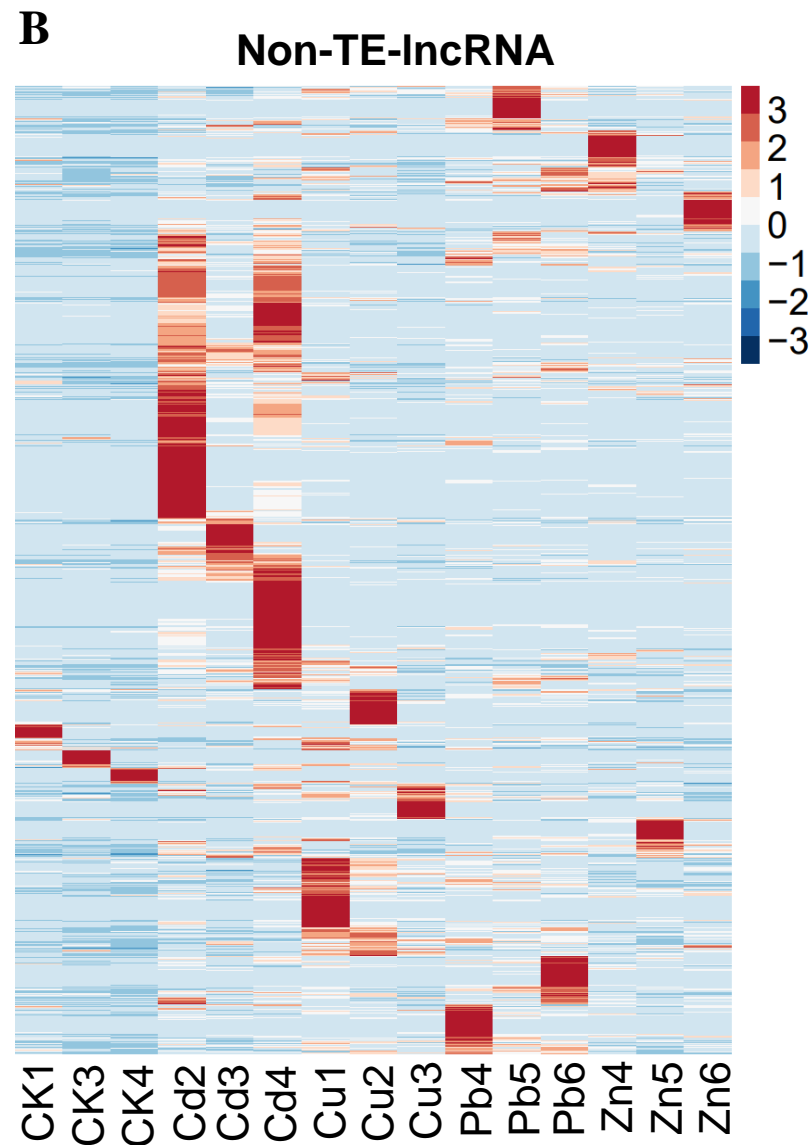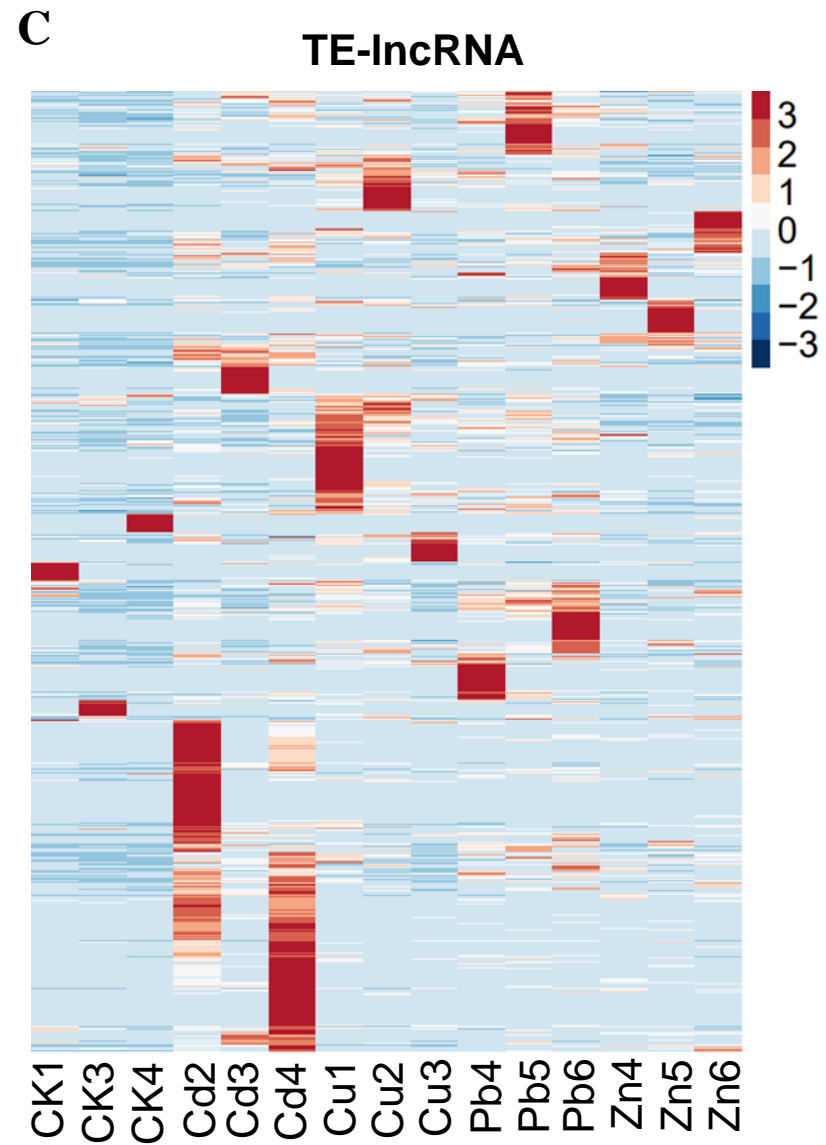

Supplement: Supplementary file 1 [file insects-15-00950-s001.zip › Figure_S4.pdf]

**A**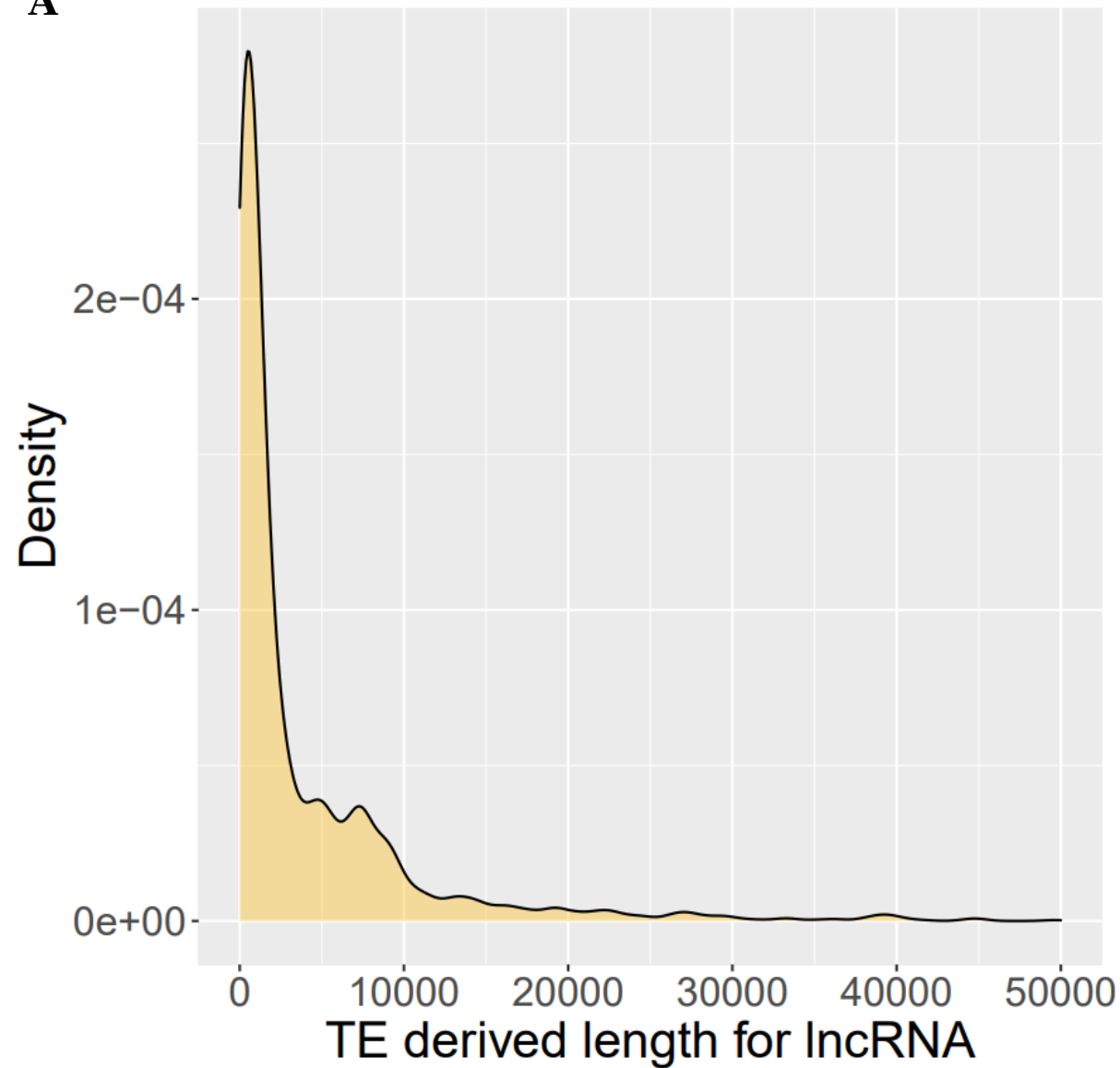**B**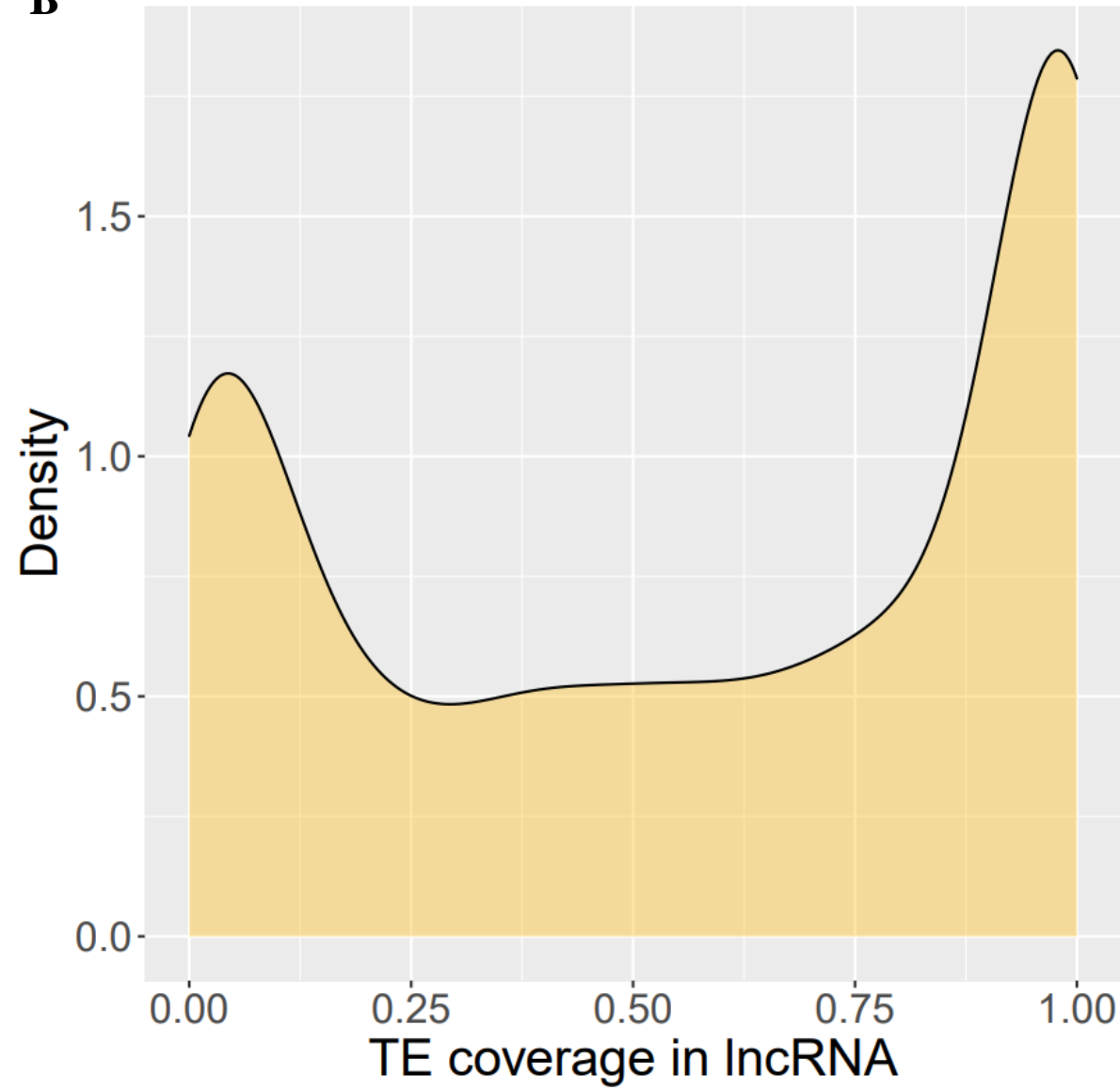

Supplement: Supplementary file 1 [file insects-15-00950-s001.zip › Figure_S7.pdf]

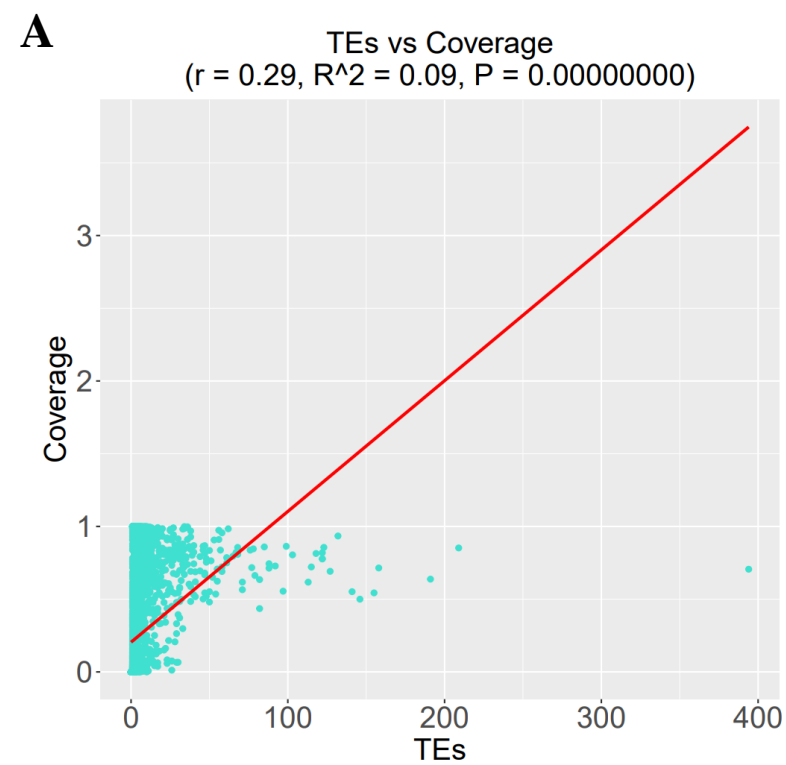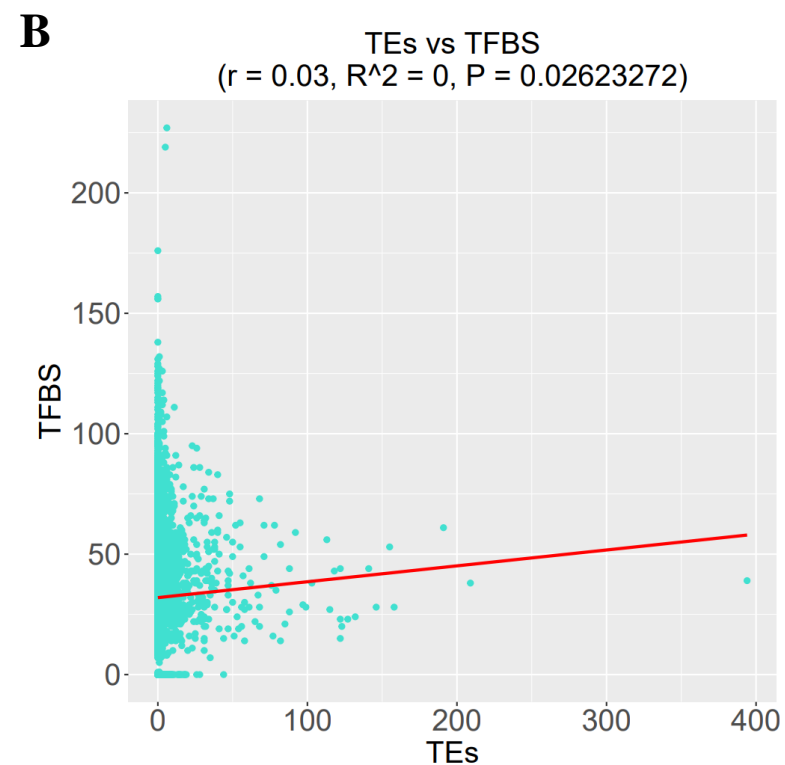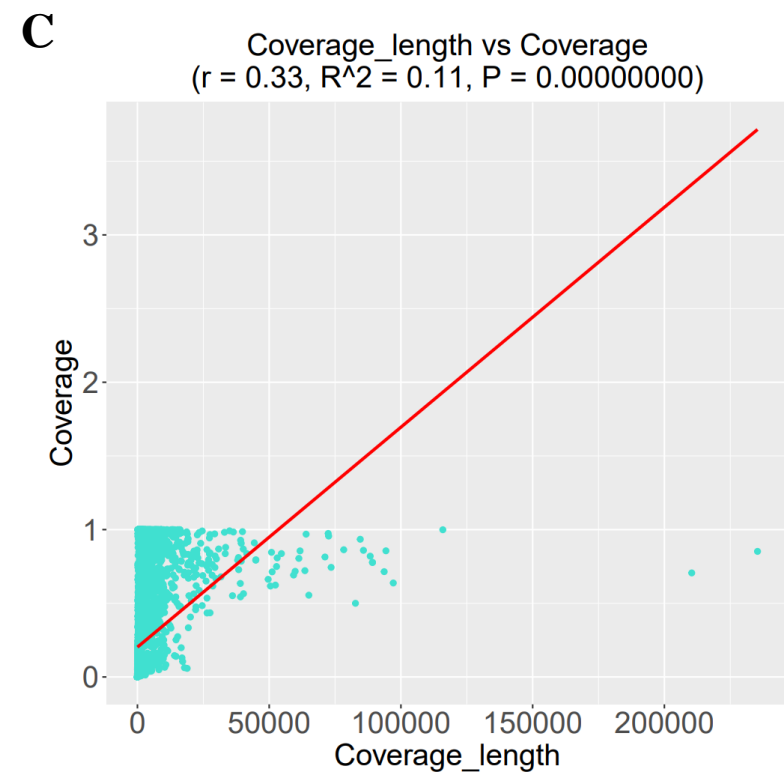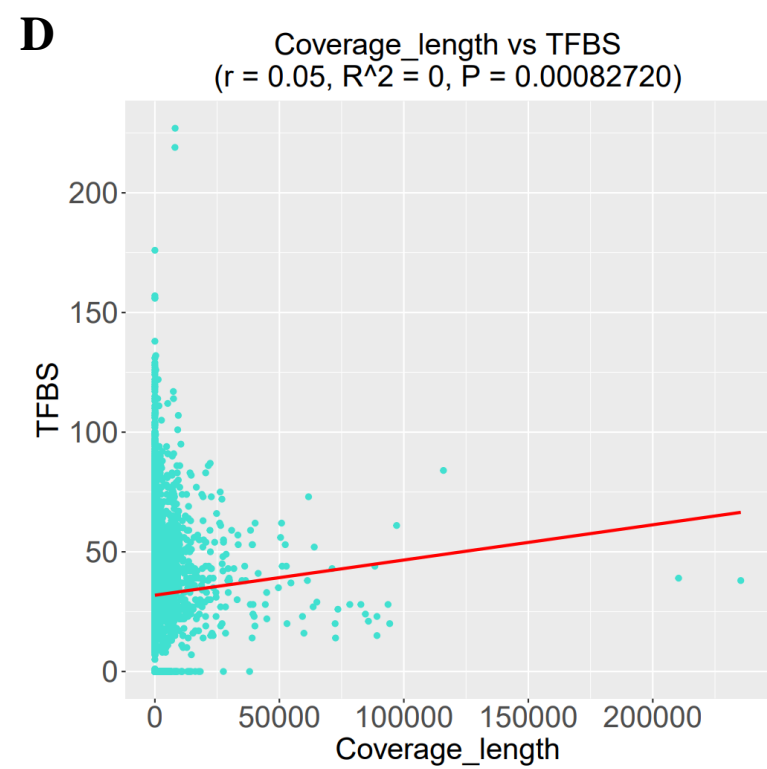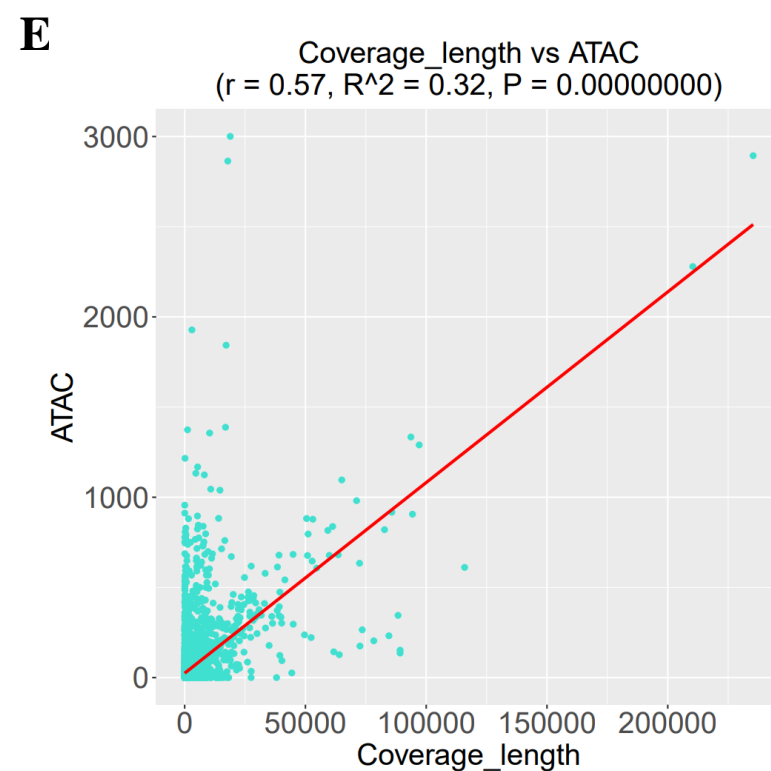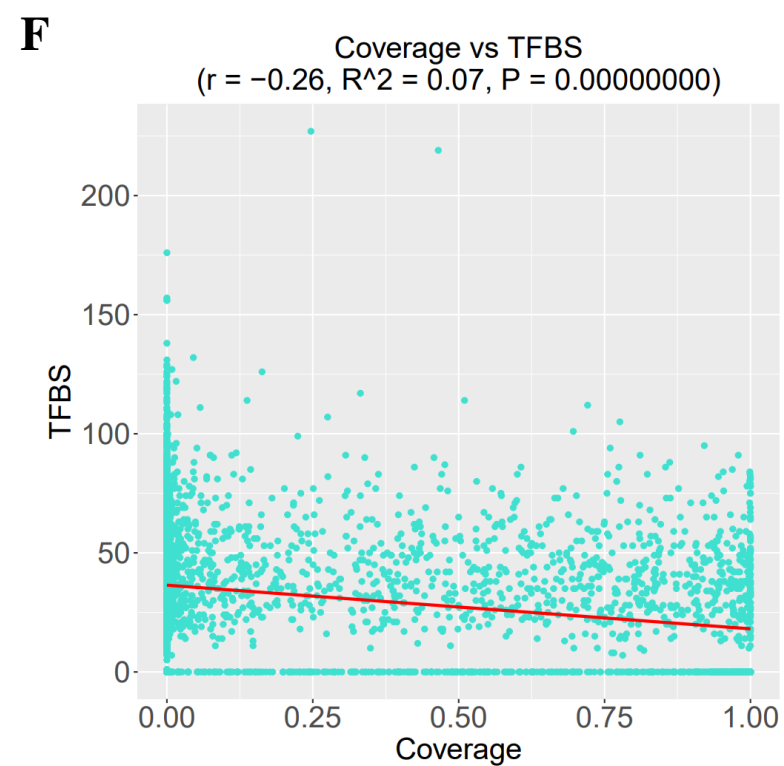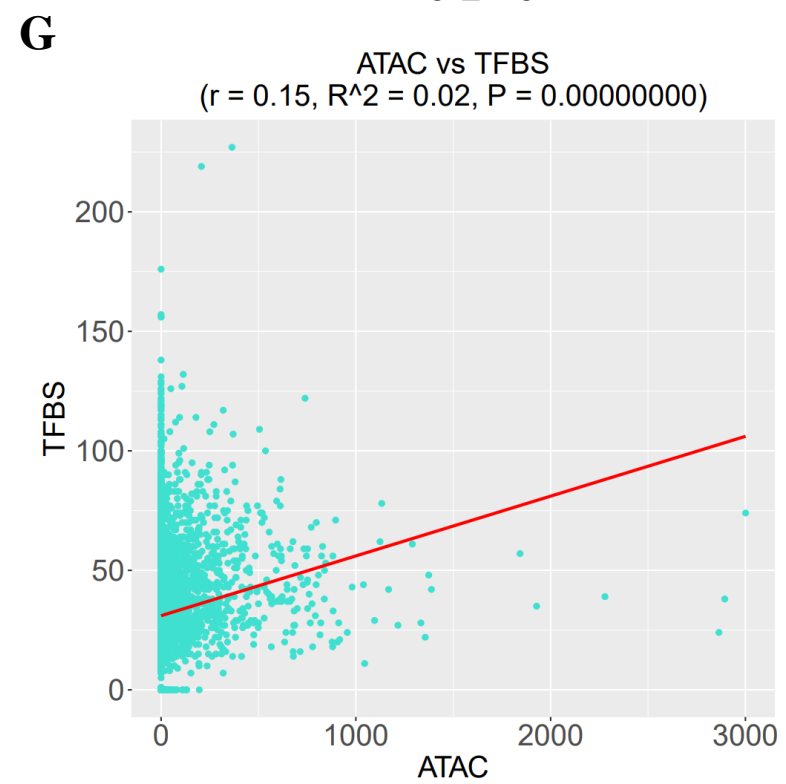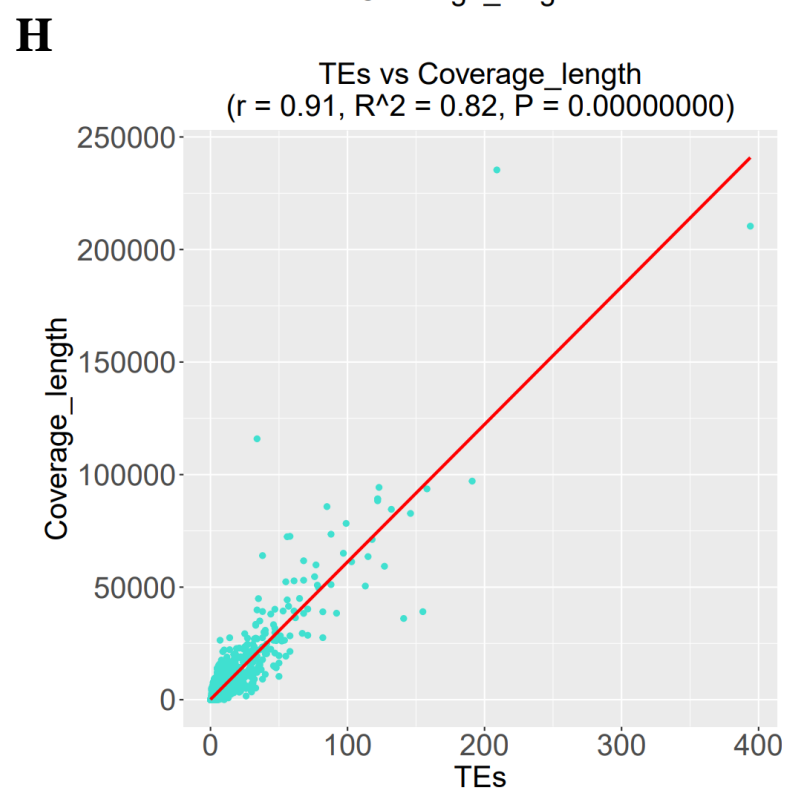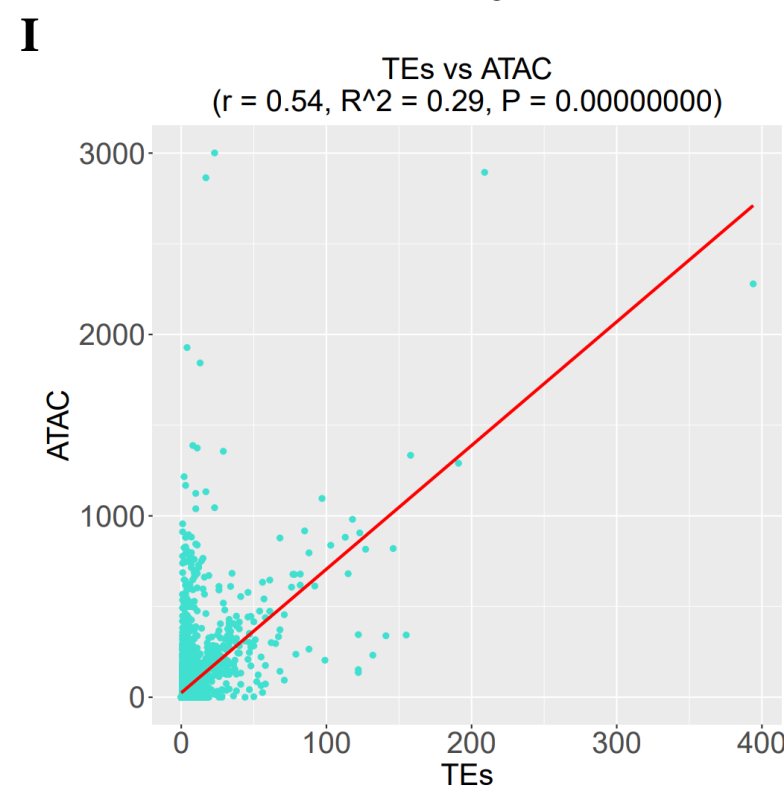

Supplement: Supplementary file 1 [file insects-15-00950-s001.zip › Figure_S8.pdf]

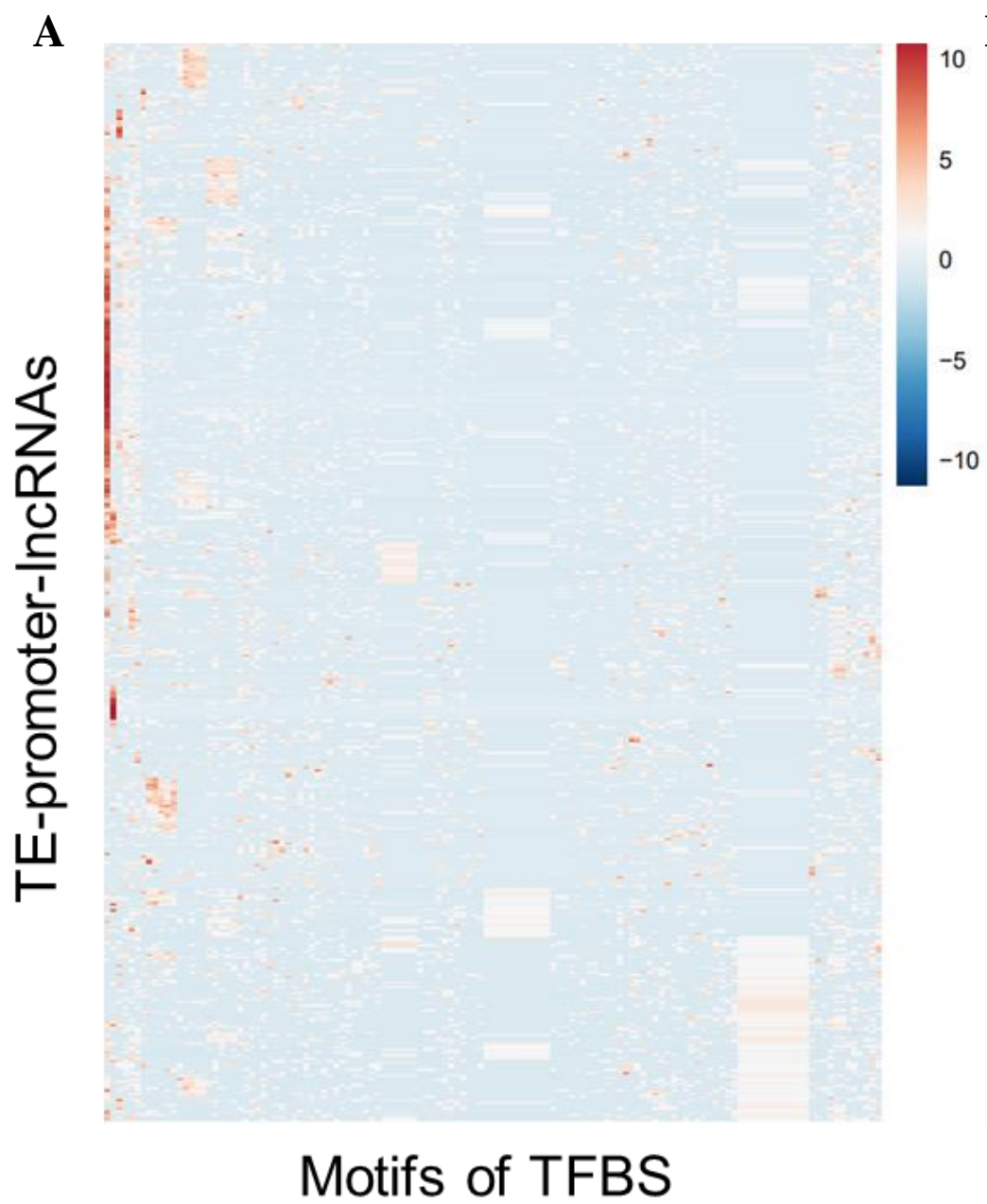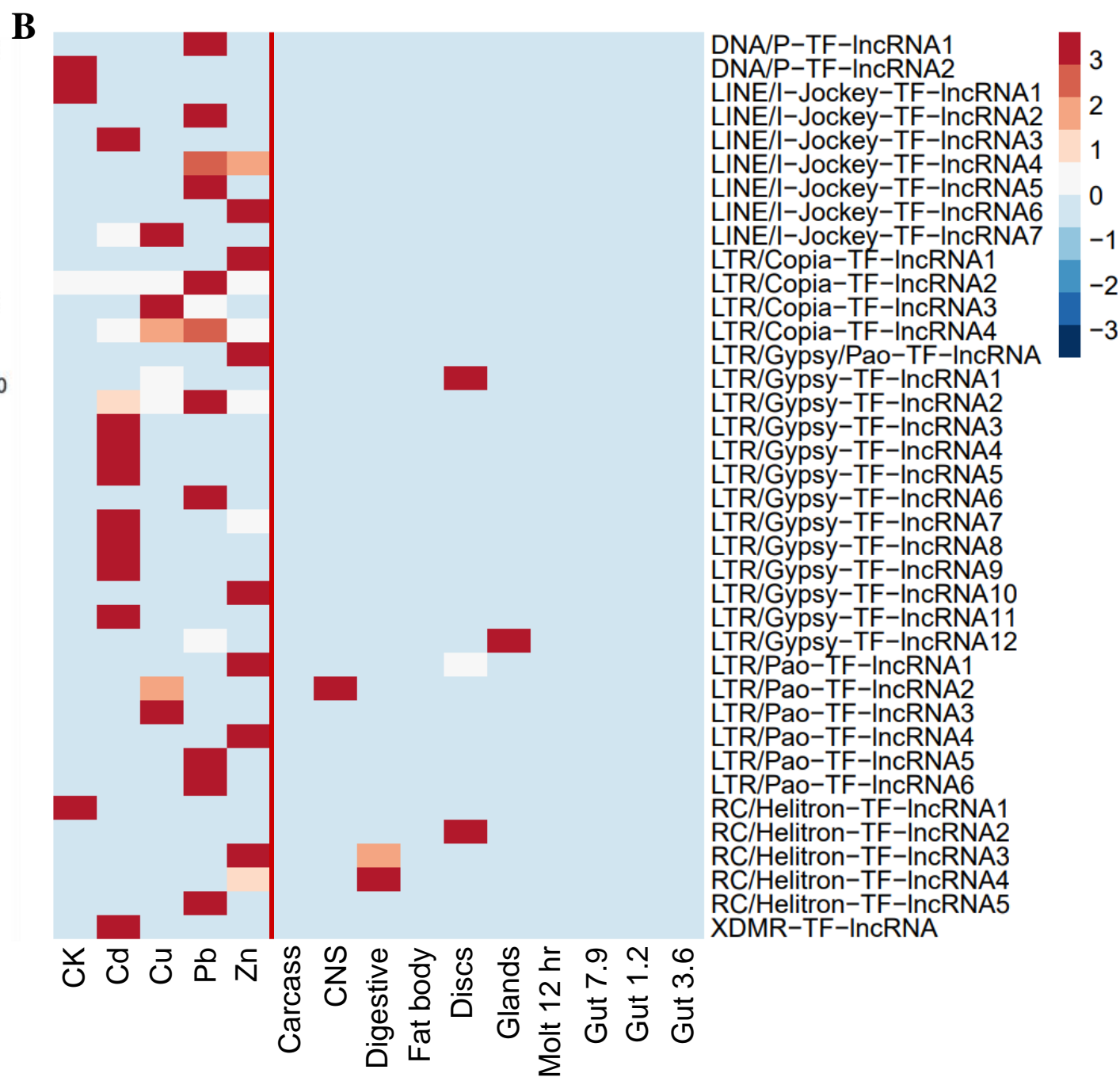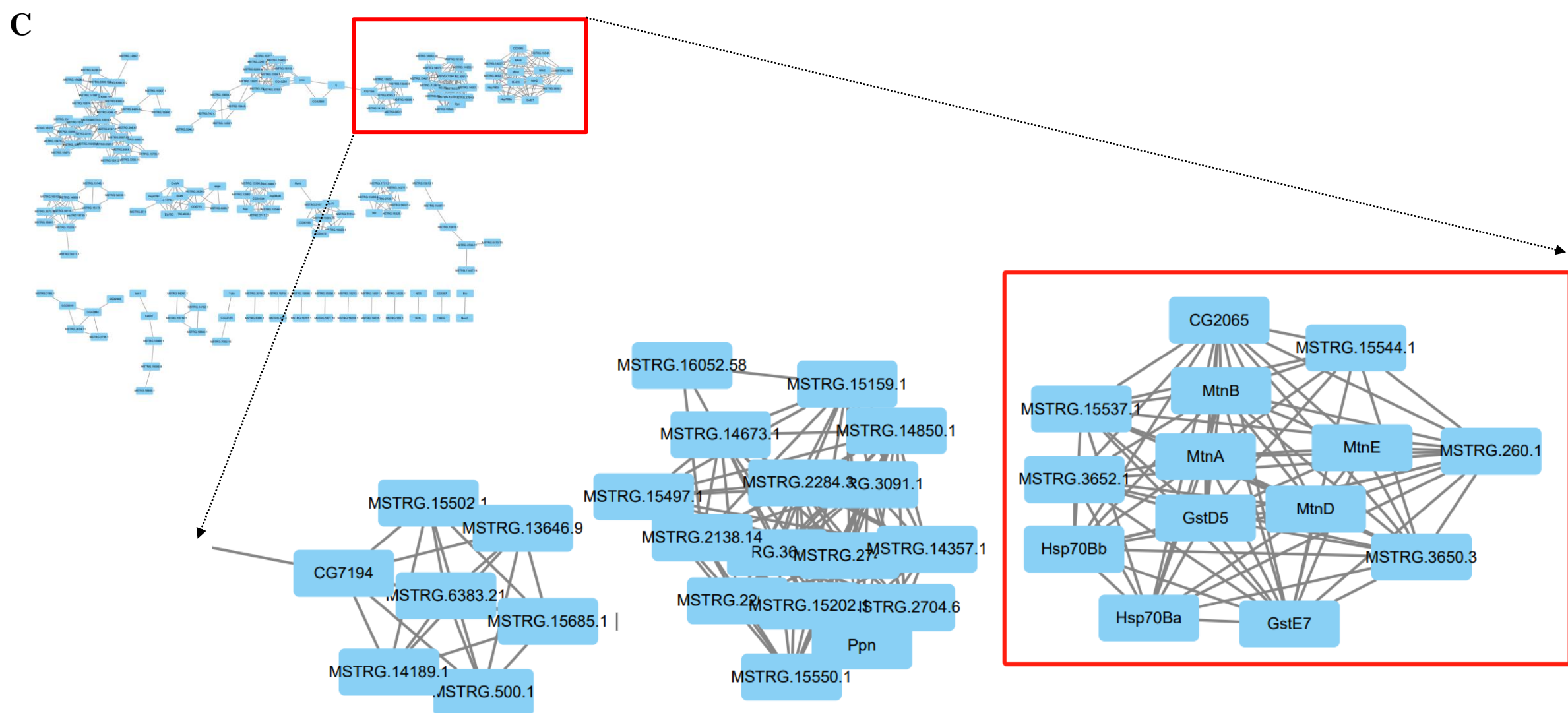

Supplement: Supplementary file 1 [file insects-15-00950-s001.zip › Figure_S9.pdf]
