# Supplementary material for: Transposable Elements Contribute to the Regulation of Long Noncoding RNAs in Drosophila melanogaster
Source: Insects. 2024 Nov 30;15(12):950. doi: 10.3390/insects15120950 (PMC11678190; doi:10.3390/insects15120950)
Supplement: Supplementary file 1 [file Figure_S5.pdf › Figure_S5.pdf]

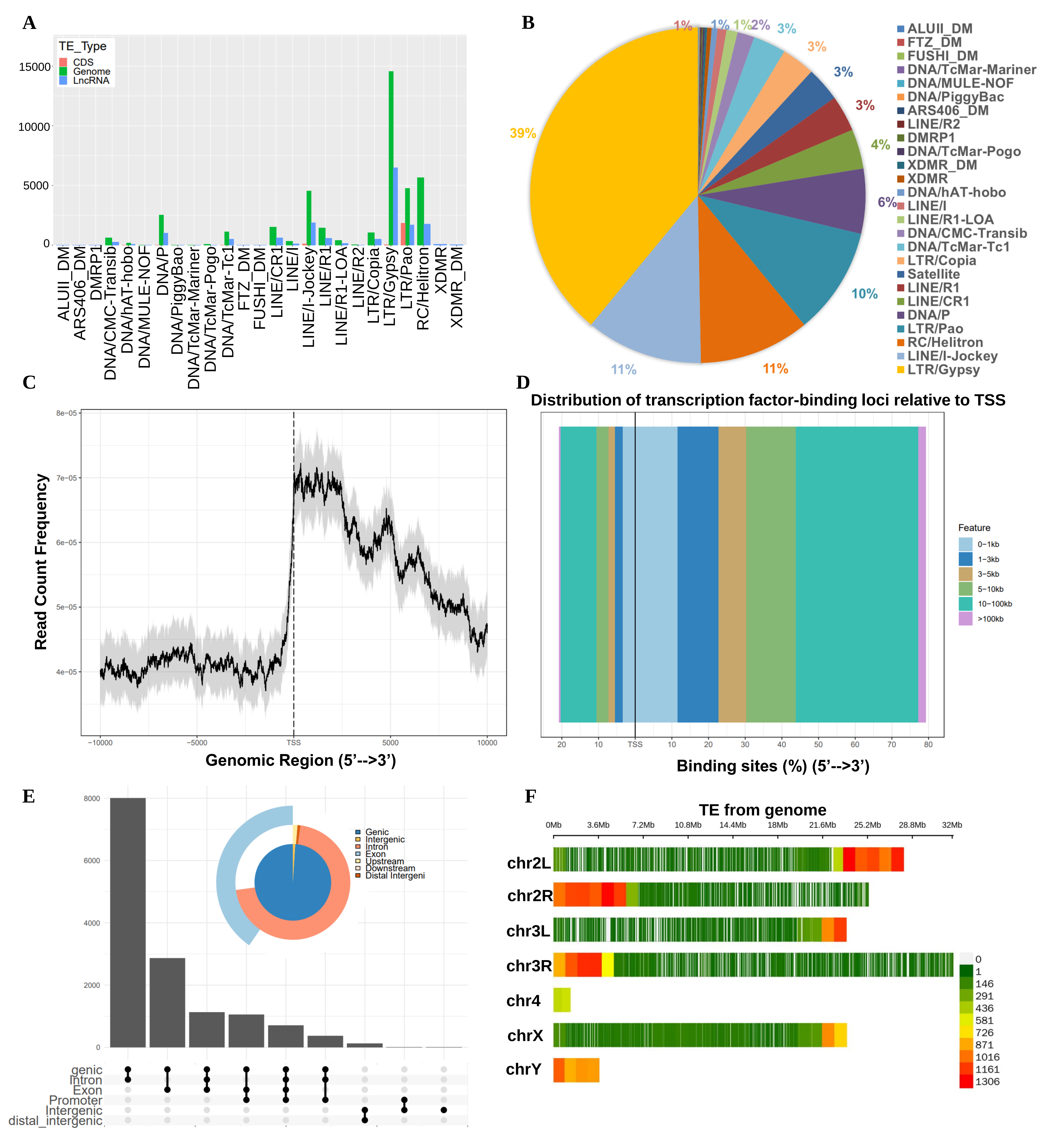
A
TE_Type
CDS
Genome
LncRNA
15000
10000
5000
0
ALUII_DM
ARS406_DM
DMRP1
DNA/CMC-Transib
DNA/hAT-hobo
DNA/MULE-NOF
DNA/P
DNA/PiggyBac
DNA/TcMar-Mariner
DNA/TcMar-Pogo
DNA/TcMar-Tc1
FTZ_DM
FUSHI_DM
LINE/CR1
LINE/I
LINE/I-Jockey
LINE/R1
LINE/R1-LOA
LINE/R2
LTR/Copia
LTR/Gypsy
LTR/Pao
RC/Helitron
XDMR
XDMR_DM
B
39%
11%
11%
10%
6%
4%
3%
3%
3%
3%
2%
1%
1%
1%
ALUII_DM
FTZ_DM
FUSHI_DM
DNA/TcMar-Mariner
DNA/MULE-NOF
DNA/PiggyBac
ARS406_DM
LINE/R2
DMRP1
DNA/TcMar-Pogo
XDMR_DM
XDMR
DNA/hAT-hobo
LINE/I
LINE/R1-LOA
DNA/CMC-Transib
DNA/TcMar-Tc1
LTR/Copia
Satellite
LINE/R1
LINE/CR1
DNA/P
LTR/Pao
RC/Helitron
LINE/I-Jockey
LTR/Gypsy
C
Read Count Frequency
8e−05
7e−05
6e−05
5e−05
4e−05
−10000
−5000
TSS
5000
10000
Genomic Region (5’-->3’)
D
Distribution of transcription factor-binding loci relative to TSS
Feature
0−1kb
1−3kb
3−5kb
5−10kb
10−100kb
>100kb
20
10
TSS
10
20
30
40
50
60
70
80
Binding sites (%) (5’-->3’)
E
8000
6000
4000
2000
0
Genic
Intergenic
Intron
Exon
Upstream
Downstream
Distal Intergeni
genic
Intron
Exon
Promoter
Intergenic
distal_intergenic
F
TE from genome
0Mb
3.6Mb
7.2Mb
10.8Mb
14.4Mb
18Mb
21.6Mb
25.2Mb
28.8Mb
32Mb
chr2L
chr2R
chr3L
chr3R
chr4
chrX
chrY
0
1
146
291
436
581
726
871
1016
1161
1306
